# An infection-microenvironment-targeted and responsive peptide-drug nanosystem for sepsis emergency by suppressing infection and inflammation

**DOI:** 10.1016/j.ajps.2023.100869

**Published:** 2023-11-28

**Authors:** Wei He, Daan Fu, Yongkang Gai, Xingxin Liu, Chang Yang, Zhilan Ye, Xu Chen, Jia Liu, Bingcheng Chang

**Affiliations:** a The Second Clinical College, The Second Affiliated Hospital, Guizhou University of Traditional Chinese Medicine, Guiyang 550003, China; b Research Center for Tissue Engineering and Regenerative Medicine, Tongji Medical College, Union Hospital, Huazhong University of Science and Technology, Wuhan 430022, China; c Department of Anesthesiology, Tongji Medical College, Union Hospital, Huazhong University of Science and Technology, Wuhan 430022, China; d Department of Laboratory Medicine, West China Second University Hospital, Sichuan University, Chengdu 610065, China; e Department of Nuclear Medicine, Hubei Province Key Laboratory of Molecular Imaging, Tongji Medical College, Union Hospital, Huazhong University of Science and Technology, Wuhan 430022, China; f Guizhou Provincial Key Laboratory of Pharmaceutics, Guizhou Medical University, Guiyang 550004, China; g Department of Geriatrics, Tongji Medical College, Union Hospital, Huazhong University of Science and Technology, Wuhan 430022, China

**Keywords:** Infectious microenvironments, Sepsis, Nanoscale drug delivery systems, Pathogens, Omiganan

## Abstract

Sepsis is a life-threatening emergency that causes millions of deaths every year due to severe infection and inflammation. Nevertheless, current therapeutic regimens are inadequate to promptly address the vast diversity of potential pathogens. Omiganan, an antimicrobial peptide, has shown promise for neutralizing endotoxins and eliminating diverse pathogens. However, its clinical application is hindered by safety and stability concerns. Herein, we present a nanoscale drug delivery system (Omi-hyd-Dex@HA NPs) that selectively targets infectious microenvironments (IMEs) and responds to specific stimuli for efficient intervention in sepsis. The system consists of omiganan-dexamethasone conjugates linked by hydrazone bonds which self-assemble into nanoparticles coated with a hyaluronic acid (HA). The HA coating not only facilitates IMEs-targeting through interaction with intercellular-adhesion-molecule-1 on inflamed endotheliocytes, but also improves the biosafety of the nanosystem and enhances drug accumulation in primary infection sites triggered by hyaluronidase. The nanoparticles release dual drugs in IMEs through pH-sensitive cleavage of hydrazone bonds to eradicate pathogens and suppress inflammation. In multiple tissue infection and sepsis animal models, Omi-hyd-Dex@HA NPs exhibited rapid source control and comprehensive inflammation reduction, thereby preventing subsequent fatal complications and significantly improving survival outcomes. The bio-responsive and self-delivering nanosystem offers a promising strategy for systemic sepsis treatment in emergencies.

## Graphical abstract

Omi-hyd-Dex@HA NPs intelligently deliver and release drugs to infected areas, controlling infection and inflammation in septic animals and enhancing survival. It is a universal and self-delivering nanosystem for sepsis emergencies.

**Figure:**
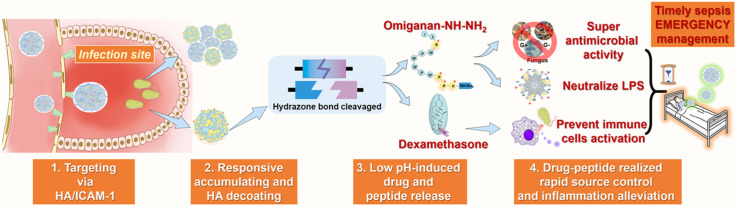

## 1 Introduction

Sepsis is a fatal condition that occurs when pathogens invade the body and trigger a dysregulated cytokine storm [1]. This leads to irreversible organ dysfunction and death, making sepsis one of the most common causes of mortality in the intensive care unit (ICU) [1,2]. In 2017, sepsis affected 48.9 million people worldwide, and 11 million sepsis-related deaths accounted for over a third of all hospital deaths [3,4]. Despite the urgency and severity of sepsis, few effective drugs are available in the clinic for its treatment [5,6]. Therefore, there is an unmet need for a novel pharmacological agent that can improve sepsis outcomes. Sepsis, caused by bacteria, fungi and viruses, presents significant challenges in identifying the pathogens involved and determining their sensitivity to drugs, which can result in missed treatment opportunities [3,[7], [8], [9]]. In these urgent situations, clinicians often resort to empirical antibiotics, particularly when drug resistance is a concern. However, this approach can also harm clinical outcomes [8], [9], [10], [11]. Moreover, the empirical overuse of antibiotics contributes to the global problem of bacterial resistance [12,13]. Additionally, in infectious microenvironments (IMEs), bacterial components such as lipopolysaccharide (LPS) and DNA are released, triggering the immune system and causing cytokine storms [14,15]. This, in turn, leads to subsequent complications such as systemic inflammatory response syndrome (SIRS) and disseminated intravascular coagulation (DIC), inflicting irreversible damage on patients [16,17]. Clinical treatments utilizing small-molecule antibiotics and anti-inflammatory drugs have limited effectiveness, mainly due to their poor targeting ability and limited availability [15]. Furthermore, the systemic administration of these drugs can lead to severe side effects [6,^13^,^18^,19]. To address these challenges, researchers have turned to the development of nanoscale drug delivery systems and artificial polymeric nanoparticles to enhance targeted drug delivery specifically to IMEs [20], [21], [22], [23], [24], [25], [26], [27]. However, the effectiveness of these systems in managing sepsis caused by unidentified pathogens remains uncertain. Additionally, the intricate structures and potential safety risks associated with these systems pose additional obstacles [9,28]. Therefore, there is a pressing need for a universally applicable therapeutic strategy to effectively control the source of infection and reduce excessive inflammation in IMEs while considering the involvement of components like LPS, as well as the complications of SIRS and DIC.

Omiganan is a potent antimicrobial peptide (AMP) derivative with broad-spectrum activity against various pathogens such as Gram-positive (G+) bacteria, Gram-negative (G-) bacteria and fungi [29,30]. Its ability to disrupt cell membranes makes it a promising candidate for addressing the issue of antibiotic resistance [31], [32], [33]. In addition, it has shown potential in inhibiting the release of endotoxins, like LPS, which triggers inflammatory responses and leads to sepsis [34], [35], [36]. By neutralizing the negative charge of LPS, omiganan can modulate its aggregation and prevent the amplification of inflammatory signals [34,37]. Through rational modifications, omiganan can be further optimized for enhanced antibacterial activity and structural functionality [33,^38^,39]. However, there are challenges in translating omiganan for clinical use, including its fast clearance from the bloodstream, and risk of hemolysis [29,^31^,40].

**Scheme 1:**
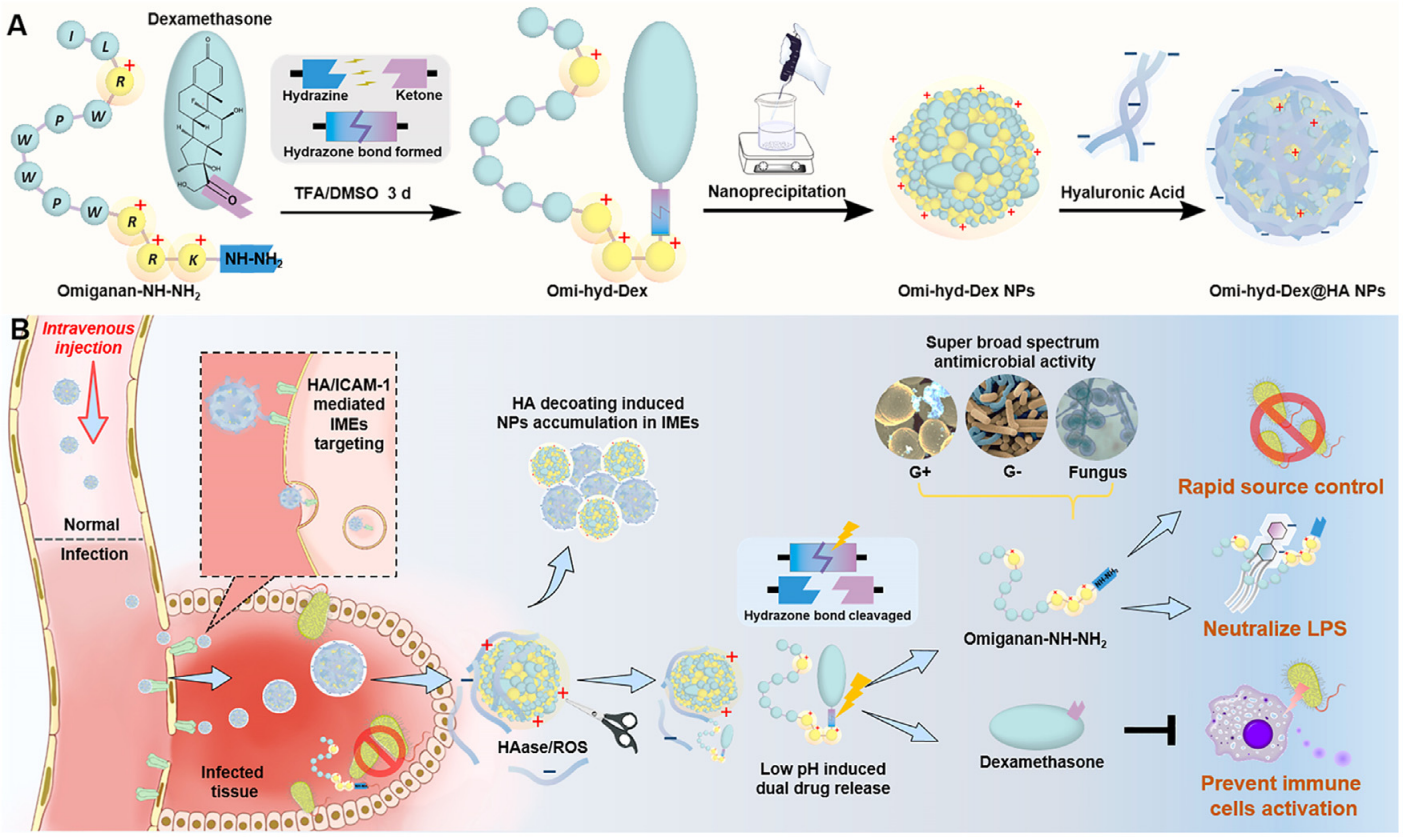
Systemic treatment of sepsis by Omi-hyd-Dex@HA NPs. (A) The preparation process of Omi-hyd-Dex@HA NPs. Omi-hyd-Dex NPs were formed by PLGA-assisted self-assembly of hydrazone-linked peptide-drug conjugates (Omi-hyd-Dex). (B) Schematic illustration of the smart dual-drug delivery approach for systemic treatment of sepsis by Omi-hyd-Dex@HA NPs. The NPs targeted the IMEs and responsively released AMP (omiganan) and anti-inflammatory agent (Dex) for the emergent treatment of sepsis caused by multiple pathogens.

To minimize the toxic side effect of omiganan and optimize its anti-inflammatory capability, we designed an IMEs-responsive self-delivery nanosystem consisting of omiganan, an anti-inflammatory agent, and natural polysaccharide to achieve on-demand degradation and responsive drug release (Scheme 1). Based on the cationic hydrophilic fragments on omiganan, we chose dexamethasone (Dex, a hydrophobic anti-inflammatory drug) to link with this peptide via a hydrazone bond to construct an amphiphilic conjugate (Omi-hyd-Dex). With the assistance of PLGA, this conjugate could self-assemble into nanoparticles (Omi-hyd-Dex NPs) in an aqueous solution without introducing any other hazardous materials. Then, the negatively-charged hyaluronic acid (HA, a natural ligand of ICAM-1 and CD44) was used to coat Omi-hyd-Dex NPs to form a core-shell structural formulation (Omi-hyd-Dex@HA NPs) [41], [42], [43]. This HA coating could not only eliminate the hemolytic activity of omiganan to reduce side effects but also act as the IMEs targeting molecule through interaction with intercellular adhesion molecule-1 (ICAM-1) on inflamed endothelial cells [42,43]. After entering IMEs, the HA coating would be degraded and detached to expose the cationic surface of the Omi-hyd-Dex core and enable it to accumulate in IMEs. Meanwhile, the hydrazone bond between omiganan and Dex could be cleaved in response to the acidic condition of IMEs, thereby releasing the cationic peptide and anti-inflammatory agent that would concurrently inhibit the infection and inflammation precisely.

Following this rational design, we synthesized an omiganan-Dex conjugate and fabricated Omi-hyd-Dex@HA NPs by PLGA-assisted self-assembly. The Omi-hyd-Dex@HA NPs preserved the bioactivities of omiganan against G+/G- bacteria and fungi infection, demonstrated potent inflammation-inhibiting and LPS-neutralizing capacity, and showed great biocompatibility *in vitro* and *in vivo*. The Omi-hyd-Dex@HA NPs could selectively accumulate in the infected tissues through HA/ICAM-1-mediated targeting effect and significantly decreased the mortality of sepsis mice by inhibiting infection and inflammation. Most importantly, Omi-hyd-Dex@HA NPs displayed remarkable lifesaving ability in the models of lethal sepsis induced by two clinical pathogenic bacteria and their respective drug-resistant strains, compared with clinically used drug combinations for sepsis. Therefore, Omi-hyd-Dex@HA NPs formulation is a promising self-delivery therapeutic nano-agent for systemic treatment of sepsis in urgent clinic front line.

## 2 Materials and methods

### 2.1 Materials

Trifluoroacetic acid (TFA), hyaluronic acid (HA; Mw 800KDa∼1.0MDa), dexamethasone (Dex), succinic anhydride (sa), 1-(3-dimethylaminopropyl)−3-ethylcarbodiimide hydrochloride (EDC), N‑hydroxy succinimide (NHS), N-hydroxysulfosuccinimide sodium salt (Sulfo-NHS), polyacrylic acid (PAA; Mw 4,500), poly(lactic-co-glycolic acid) (Resomer®RG 504, Mw 38,000–54,000), lipopolysaccharides (LPS; *Escherichia coli*. O111:B4) and 3-(4,5-dimethylthiazol-2yl)−2,5-diphenyl tetrazolium bromide (MTT) were purchased from Aladdin Co. Ltd (Shanghai, China). 1,1′-Dioctadecyl-3,3,3′,3′-tetramethylindocarbocyanine perchlorate (DiI), 1,1′-dioctadecyl-3,3,3′,3′-tetramethylindotricarbocyanine iodide (DiR), FITC-PEG_4—_COOH was obtained from Xian Ruixi Biological Technology Co. Ltd. Omiganan-NH—NH_2_ (ILRWPWWPWRRK-NH—NH_2_; C_90_H_128_N_28_O_12_; MW:1794.18) was designed by our team and constructed by solid-phase synthesis method. Omiganan-NH_2_ (as a control group) was purchased from MedChemExpress Ltd. The LIVE/DEAD BacLight Bacterial Viability Kit (L7012) was purchased from Invitrogen (USA). Vancomycin, levofloxacin, imipenem, and moxifloxacin were purchased from Innochem (China). Lipopolysaccharides (LPS; *E. coli*. 055:B5) were provided by Solarbio Co. Ltd (China). Hyaluronidase (HAase BR, 300–500 u/mg; S10060) was purchased from Shanghai Yuanye Bio-Technology Co. Ltd. Dimethyl sulfoxide (DMSO), acetone, phenol, trichloroacetic acid, ethanol, and hydrochloric acid were provided by Sinopharm Group Co. Ltd (China). All the reagents were analytical reagent grade and used without further purification.

### 2.2 Cell lines, bacterial strains, and animals

Human umbilical vein endothelial cells (HUVECs) and Raw 264.7 macrophages were generously provided by Union Hospital of Huazhong University of Science and Technology and cultured with 10% fetal bovine serum (FBS), 100 unit/ml penicillin and 100 µg/ml streptomycin in RPMI 1640 medium at 37 °C. Mouse primary microvascular endothelial cells were isolated by our group from BALB/c mice and cultured in RPMI 1640 (10% FBS) with 100 unit/ml penicillin and 100 µg/ml streptomycin at 37 °C. Bacterial strains including *S. aureus* (ATCC 29,213), Methicillin-resistant *S. aureus* (MRSA), *P. aeruginosa, Klebsiella pneumoniae (K.P.)*, Multidrug-resistant *K. pneum*oniae (MDR-*K.P.*), *Candida albicans* were kindly provided by Departments of Basic medicine or clinically isolated in the Second Affiliated Hospital of Guizhou University of Traditional Chinese Medicine. Female Kunming mice (7 to 10 weeks old, 25–35 g) and rats (8 weeks old) were provided by the Laboratory Animal Center at Guizhou University of Traditional Chinese Medicine (Guizhou, China) and Charles River Laboratories (Beijing, China). Female BALB/c mice (6 to 8 weeks old, 20–25 g) were purchased from the Laboratory Animal Center at Huazhong University of Science and Technology (Wuhan, China). The animals were allowed to access food and water freely under the condition of no specific pathogens (SPF). The animal experiments followed the European Community guidelines and the standards of care and use of laboratory animals approved by the Laboratory Animal Ethics Committee of the Second Affiliated Hospital of Guizhou University of Traditional Chinese Medicine.

### 2.3 Synthesis and characterization of Omi-hyd-Dex and Omi-sa-Dex

Omiganan-NH—NH_2_ was designed by our team and constructed by solid-phase synthesis method by GL Biochem Ltd. Equimolar quantities of Omiganan-NH—NH_2_ (179.42 mg) and Dex (39.24 mg) were dissolved in DMSO (10 ml). During sustained stirring at room temperature, TFA (100 µl) was added to the solution. Two days later, the mixture was purified by the acetone precipitation method. The collected Omi-hyd-Dex conjugate was further dialyzed (MWCO 1,000 Da) in diH_2_O and freeze-dried for MS (6125B, ESI, Agilent or Microflex, MALDI-TOF, Bruker Daltonics) and ^1^HNMR (*d6*-DMSO, 600 MHz, Bruker) identification.

Dex (2 g) and DIPEA (1 ml) were added into 50 ml DCM under 800 rpm stirring. Then, sa (1 g) was added to the solution being heated at 40 °C under reflux for 24 h. Dex-succinate-COOH (Dex-sa-COOH) was obtained by silica gel chromatography purification. Last, Dex-succinate-COOH was conjugated to omiganan through amino residue on lysine. Dex-succinate-COOH (48.86 mg) and omiganan (177.91 mg) were put into 20 ml DMSO, followed by adding EDC/NHS (191.7 mg/11.51 mg) for catalyzing. After 12 h, the reaction was stopped and the product Omi-sa-Dex was purified by the same procedures as Omi-hyd-Dex was obtained. The produced Omi-sa-Dex conjugate was further verified by MS.

### 2.4 Preparation and characterization of Omi-hyd-Dex@HA NPs

Omi-hyd-Dex@HA NPs were prepared by the nanoprecipitation method with slight modification. Omi-hyd-Dex (10 mg) and PLGA (2 mg; Mw 38,000–54,000 Da) were dissolved in 1 ml DMSO (0.5 mg DiR was added into DMSO solution to produce DiR-labeled NPs). Under intense stirring, 15 ml diH_2_O (0.4% PVA, m/v) was added into the DMSO solution drop by drop. After 60 min, another 20 ml diH_2_O containing 5 mg HA was added for coating (1 mg FITC-PEG-COOH was added into this solution to produce FITC-labeled NPs). The stable Omi-hyd-Dex@HA NPs were produced by adding 2 mg EDC and 0.5 mg Sulfo-NHS for building covalent cross-linking between HA and core NPs. After 4 h, the resulting suspension was centrifuged at 15,000 rpm for 10 min and resuspended in PBS. Omi-hyd-Dex@HA NPs were obtained for the following experiment. Omi-sa-Dex@HA NPs were also constructed by Omi-sa-Dex conjugate in a similar procedure as Omi-hyd-Dex@HA NPs.

All NPs were suspended in PBS (pH 6.0 or 7.4) buffer with or without HAase. Particle sizes, polydispersity indexes and zeta potential measurements were executed on Malvern Zetasizer Nano ZS90 or Brookhaven NanoBrook 90plus. And the morphology of NPs was observed through the Hitachi TEM system with 2% phosphotungstic acid for negative staining.

### 2.5 Stability, drug loading capacity, and drug release profiles testing

Omi-hyd-Dex@HA NPs were suspended in PBS (20% FBS) at 37 °C on a shaking bath to mimic the condition in blood circulation. At regular time points, the Omi-hyd-Dex@HA NPs suspension was taken out. After ultrasound dispersing, particle size was monitored.

The drug loading content in Omi-hyd-Dex@HA NPs was determined by HPLC (LC-20, Ashimadzu) and HPLC-MS (Ultimate 3000 UHPLC-Q Exactive, Thermofisher). Omi-hyd-Dex@HA NPs were weighed and dissolved in DMSO (containing 1% HCl, m/v). After 30 min, the solution was centrifuged for separating the supernatant, which was diluted with methanol for further analysis. Dex loading content was monitored by HPLC equipped with Diamond C_18_ column (mobile phase: methanol/water, 1.0 ml/min; wavelength 240 nm). Omiganan-NH—NH_2_ loading content was monitored by HPLC-MS equipped with a Gemini-NX C_18_ 110A column (mobile phase: acetonitrile/water with 0.1% TFA, 1.0 ml/min). Drug content was calculated by establishing standard curves with standard substances. The drug loading mass proportion was calculated and plotted.

Drug release profiles of Omi-hyd-Dex@HA NPs and Omi-sa-Dex@HA NPs were evaluated by continuous supernatant separation. NPs (0.5 mg) were incubated in the buffers (PBS pH 7.4 and 6.0, 1.5 ml) with or without HAase. The NPs suspension was shaken at 200 rpm. At certain time points, NPs suspension was centrifuged at 8,000 rpm for 10 min to separate the supernatant, and the media was renewed. The concentrations of Omiganan-NH—NH_2_ and Dex in the collected media were examined and calculated through HPLC and HPLC-MS analysis as above described.

### 2.6 Biosafety of Omi-hyd-Dex@HA NPs

Cell membrane destruction effect was observed on Raw264.7 macrophages using a confocal laser scanning microscope image system. Cells were seeded in glass-bottom microplates (YA0570, Solarbio) at a density of 1 × 10^7^ cells/ml. PBS, Omiganan-NH—NH_2_ (DMSO), or Omi-hyd-Dex@HA NPs (peptide equivalent, 100 µg/ml) were added into cell culture media for 12 or 24 h incubation. After the media was removed, cells were stained with Hoechst 33342 (1 µM) and free DiI (2 µM) successively for 10 min each. Then, cells were imaged using a Nikon Ti-U microscope equipped and an EM-CCD camera (iXon+; Andor) for morphological observation.

Hemolysis testing of Omiganan-NH—NH_2_ or NPs was carried out as described in our previous article [44]. Fresh blood was donated by our healthy laboratory workers. After two times of hypothermic normal saline solution washing, erythrocytes were collected by centrifuge (800 rpm, 5 min) and re-suspended in PBS (2%, v/v). Erythrocytes suspension was mixed with PBS, Omiganan-NH—NH_2,_ or NPs (peptide concentration 10 to 160 µg/ml) for 5-h incubation at 37 °C. All samples were centrifuged at 800 rpm and imaged. Then, the supernatant of each group was separated and tested under a UV–Vis spectrophotometer (absorbance, 545 nm; V 5600, Metash Instruments Co.Ltd, Shanghai).

Cytotoxicity of Omiganan-NH—NH_2_ and NPs was assessed with HUVECs by MTT assay. HUVECs were suspended in RPMI-1640 (10% FBS) and seeded in 96-well plates (10^4^ cells/well) for 12 h. Then, different concentrations of Omiganan-NH—NH_2_ or NPs were mixed with fresh culture media for further 24 h incubation. Next, culture media was exchanged with MTT-containing cell culture media (5 mg/ml) for a 4 h reaction. After the supernatant was carefully removed, the formazan was dissolved in DMSO (150 µl) and tested by a microplate reader (Infinite M200, Tecan, Switzerland).

Female BALB/c mice were used in testing the biosafety of Omiganan-NH—NH_2_ or NPs (peptide equivalent, 10 mg/kg) *in vivo* through blood chemistry analysis. Twelve mice were randomly divided into three groups and treated with four doses of PBS, Omiganan-NH—NH_2_ (DMSO), or Omi-hyd-Dex@HA NPs (every 2 d). One day after the final injection, the mice were euthanized and the blood was obtained. The main organs, including the heart, liver, spleen, lung, and kidney, were taken out and fixed for hematoxylin and eosin (H&E) staining analysis. All slide photos were obtained through the Olympus BX51 microscope.

### 2.7 Cellular uptake and penetration of Omi-hyd-Dex@HA NPs

TNF-α-activated HUVECs were employed to study the Omi-hyd-Dex@HA NPs binding and cellular uptake profiles. HUVECs were seeded in glass-bottom microplates (YA0570, Solarbio) at a density of 1 × 10^5^ cells/ml. For activating HUVECs, 50 ng/ml of TNF-α was added into the culture media before 24 h incubation. The activated cells were washed and incubated in PBS with or without anti-CD44, anti-ICAM-1, or free HA for 1 h at 37 °C. After PBS washing, FITC-labeled NPs (10 µg/ml) were added for further 4-h incubation. Cells were washed and stained with Hoechst 33342 (1 µM) for 10 min. After 3 times of PBS washing, cells were observed and imaged by a confocal system as above described.

The inflamed endothelial penetration ability of Omi-hyd-Dex@HA NPs was assessed by Transwell assay. HUVECs were seeded in the Transwell insert wells (6.5 mm diameter, 3 µm pore size, PTFE membrane, Corning) at a density of 1 × 10^6^ cells/well for 6-h incubation. Then PBS or 50 ng/ml of TNF-α was added in. After 24 h, FITC-labeled NPs (peptide equivalent, 10 µg/ml) were suspended in 100 µl RPMI-1640 and added into the insert wells. After 16 h, the culture media in the bottom were taken out for fluorescence intensity analysis.

### 2.8 Biodistribution of Omi-hyd-Dex@HA NPs in vivo

*K.P.*-induced pneumonia models were established by the intratracheal instillation method. Female Kunming mice were anesthetized with 2% pentobarbital and placed in a supine position. Then 1 × 10^6^ CFU (Colony Forming Unit) of *K.P.* was intratracheally administrated drop by drop through a 22 G Y integrated catheter. One day later, mice were divided into 3 groups randomly and intravenously injected with PBS, DiR-labeled NPs (peptide equivalent, 5 mg/kg). After 6 or 24 h, three mice of each group were sacrificed and the main organs (heart, liver, spleen, lung and kidney) were taken out for IVIS imaging (Ex/Em = 748/780, Bruker *In-Vivo* Xtreme), and quantitative analysis.

*S. aureus*-induced infection models were established by the footpad injection method. Female BALB/c mice were anesthetized with 2% pentobarbital and placed in a supine position. Left hind limb footpad was sterilized by an iodophor tampon. Then, 100 µl *S. aureus* PBS suspension (1 × 10^6^ CFU) was subcutaneously injected into the left hind footpad. One day later, the mice were divided into 3 groups randomly and intravenously injected with PBS, DiR-labeled NPs (peptide equivalent, 5 mg/kg). After 6 or 24 h, three mice of each group were anesthetized and placed in a supine position for IVIS imaging as above and quantitative analysis.

### 2.9 Antibacterial activity of Omi-hyd-Dex@HA NPs in vitro

*In vitro,* antimicrobial efficiency was verified by minimum inhibitory concentrations (MICs) testing. MIC was defined as the lowest concentration of the drugs that inhibited bacterial growth by more than 90%. Bacteria were cultured to mid-log phase in Mueller-Hinton Broth (BD Difco.) media and diluted to 1 × 10^6^ CFU/ml. A series of drugs or NPs solutions/suspensions (drugs or peptides concentration 0.5–128 µg/ml) with 5 × 10^5^ CFU/ml bacteria were prepared on 96-well microplates for 18 h incubation at 37 °C. The absorbance (600 nm) of cultures in wells was measured and plotted by a microplate reader (Infinite F200; TECAN). Tests of each drug were repeated three times.

Bio-SEM was employed to observe the morphologies of *K.P*. (1 × 10^5^ CFU/ml) after treatment of PBS, Omiganan-NH—NH_2_ (2× MIC), or Omi-hyd-Dex@HA NPs (2× MIC) for 4 h at room temperature. Bacteria were gathered by centrifuge (1,000 rpm; 5 min) and fixed with 3% glutaraldehyde solution at 4 °C for 24 h. After ethanol dehydration and vacuum drying procedures, bacteria samples were coated with gold (Hitachi, MC1000) for SEM (Hitachi, RegulusSU8100) observation.

LIVE/DEAD bacterial viability kit (BacLight L13152, Invitrogen) was employed to investigate *K.P.* membrane permeability changes after PBS, Omiganan-NH—NH_2_ (2× MIC), or Omi-hyd-Dex@HA NPs (2× MIC) treatment. *K.P*. (1 × 10^5^ CFU/ml) was grown into a mid-log phase in Mueller-Hinton Broth (BD Difco.), washed and diluted into PBS which contained NPs or Omiganan-NH—NH_2_ for 1 h incubation. After being stained with Syto 9 and PI for 10 min as manually instructed, bacteria were smeared on a glass slide for confocal imaging.

Time-kill kinetics of all NPs and drug solutions against MDR-*K.P.* and *K.P.* were investigated by CFU assay. Bacteria were cultured to mid-log phase in Mueller-Hinton Broth (BD Difco.) media and diluted with PBS when OD_600_ = 0.30. Then, in PBS, different concentrations of NPs or drug solutions were added to the bacteria suspension. After the addition of different formulations, parts of culture media in each group were removed at specific time points for CFU enumeration via plate culture (16–18 h). The results were counted and presented by Log10 (CFU/ml).

### 2.10 LPS neutralization activity of Omi-hyd-Dex@HA NPs in vitro

LPS aggregates preparation: purchased LPS (from *E. coli.* 0111:B4) was re-purified by phenol and trichloroacetic acid. LPS was dispersed in pH 7.4 PBS (1 µM) by applying a pulsed ultrasound. Then, the LPS dispersion liquid was heated to 56 °C with continuous ultrasound for 10 min and transferred to an ice-water bath for 10 min. This step was repeated three times. LPS aggregates were obtained.

Acid-treated Omi-hyd-Dex@HA NPs were obtained by dissolving Omi-hyd-Dex@HA NPs in DMSO (containing 1% HCl, m/v) for 30 min. Then, DMSO (vehicle), different concentrations of acid-treated Omi-hyd-Dex@HA NPs or Omiganan-NH—NH_2_ (DMSO dissolved) were mixed with LPS aggregates at 37 °C with ultrasound for 10 min. The LPS aggregate size and Zeta potential changes were recorded by Brookhaven NanoBrook 90plus.

Mouse primary microvascular endothelial cells were seeded in glass-bottom microplates at a density of 10^6^ cells/ml. DMSO (vehicle), different concentrations of acid-treated Omi-hyd-Dex@HA NPs or Omiganan-NH—NH_2_ were mixed with LPS in cell culture media (without FBS) for 12 h. Cells were harvested and fixed with 4% paraformaldehyde. Subsequently, cells were washed and blocked with BSA for rat anti-mouse E-selectin antibody (100:1; UZ5, MA1–06,505, Invitrogen) incubation. Followed by FITC-labeled rabbit anti-rat IgG incubation and staining with Hoechst 33342 (1 µM) for 10 min, the fluorescence images were taken on a confocal image system as described above.

Intracellular nitric oxide (NO) production was monitored by Micro NO Content Assay Kit (Solarbio, BC1475). Raw264.7 macrophages were seeded in 6-well cell culture plates at a density of 10^7^ cells/ml. DMSO (vehicle), different concentrations of acid-treated Omi-hyd-Dex@HA NPs or Omiganan-NH—NH_2_ were mixed with LPS in cell culture media (without FBS) for 24 h. The amount of NO produced by untreated cells was also recorded as blank control. Cell media of all groups were mixed in Griess reagent (50 µl). Following instruction, absorption at wavelength 548 nm was recorded. The NO production was calculated by the standard curve method.

### 2.11 Therapeutic efficacy in LPS-induced sepsis mouse models

Female Kunming mice (body weight > 23 g) were challenged with an intraperitoneal injection of LPS solution (50 mg/kg). Mice were randomly divided into different groups. After 2 h, PBS, free (Omi/Dex), PLGA(Omi)@HA NPs, PLGA(Dex)@HA NPs, Omi-hyd-Dex@HA NPs, Omi-hyd-Dex@PAA NPs (peptide concentration equivalent to 5 mg/kg; Dex concentration equivalent to 1.1 mg/kg) was intravenously injected. To evaluate the total survival rate, all the mice were treated as described above (*n* = 9) and observed. Dead mice were removed every 12 h. The body temperature after injection at 0 and 24 h were recorded. Whole blood was collected from the mouse orbit 24 h after treatment for blood cell counting and classification (Sysmex-XN1000, Japan). Meanwhile, 2 ml PBS was intraperitoneally injected and 0.5–0.7 ml peritoneal fluid was collected soon afterward. After neutrophil counting, pro-inflammatory cytokines (TNF-α, IL-6, IL-1β) in the peritoneal fluid were quantified following manufacturer instructions on ELISA kits (CSB-E04741m, CSB-E04639m, CSB-E08054m; CUSABIO, China).

### 2.12 Therapeutic efficacy in CLP sepsis models

To evaluate the therapeutic efficacy against polymicrobial sepsis, cecal ligation and puncture (CLP) sepsis models were employed. Eight-week-old female Kunming mice were randomly divided into 6 groups (*n* = 12). After hair removal and disinfection of the abdomen, mice were anesthetized with 2% pentobarbital sodium. A midline laparotomy was operated for cecum exteriorization. Then 30% of the full length of the cecum was ligated and punctured with a 26 G needle for feces leakage. Feces and cecum were returned carefully into the abdomen afterward before the surgical incision was stitched. Mice were placed on an electric heating blanket for recovery. All model-establishing operations were finished within 2 h. PBS, free (Omi/Dex), PLGA(Omi)@HA NPs, PLGA(Dex)@HA NPs, Omi-hyd-Dex@HA NPs, Omi-hyd-Dex@PAA NPs (peptide concentration equivalent to 5 mg/kg; Dex concentration equivalent to 1.1 mg/kg) was intravenously injected 4 times within 6 d (interval of 2 d). To evaluate the total survival, all the mice were subjected to the treatment as mentioned above (*n* = 9) and observed. Dead mice were removed every 24 h. Blood and peritoneal fluid were collected as above described 24 h after the first injection. d-Dimer and fibrinogen degradation products (FDP) in blood were quantified by ELISA kits (ml038012, ml037259; MLBIO, China). Pro-inflammatory cytokines (TNF-α, IL-6, IL-1β) in the peritoneal fluid were quantified as described above. Besides, bacteria number in the peritoneal fluid was quantified by CFU assay with brain heart infusion (BHI) agar plates in aerobic and anaerobic conditions.

### 2.13 Survival rate assay of sepsis models induced by intraperitoneal injection of bacteria

To evaluate the therapeutic efficacy against sepsis caused by several clinically common pathogens, intraperitoneally injected bacteria-induced sepsis models were employed. Eight-week-old female Kunming mice were injected intraperitoneally with 1 × 10^7^ CFU of bacteria (*G*+ *S. aureus* and G- *K.P*. as well as their respective drug-resistant strains MRSA and MDR-*K.P.*) suspended in 100 µl of 1× PBS. After 2 h, mice were randomly divided into 6 groups (*n* = 9), and each mouse was injected with different therapeutics 4 times each day to simulate the clinic regimen. Mice were monitored and dead ones were taken out every 24 h.

### 2.14 Statistical analysis

All data was performed using GraphPad Prism and presented as mean ± SD. Statistical analysis was conducted using two-way ANOVA with a confidence interval of 95% (ANOVA). A *P value<0.05 was considered significant.

## 3 Results and discussion

### 3.1 The preparation and characterization of Omi-hyd-Dex@HA NPs

The Omi-hyd-Dex@HA NPs were fabricated by four steps (Scheme 1A): (1) The omiganan peptide was synthesized by solid-phase synthesis, and the C terminal was modified with hydrazine to obtain Omiganan-NH—NH_2_; (2) Omiganan-NH—NH_2_ was conjugated with Dex through hydrazone bond to obtain Omi-hyd-Dex; (3) The Omi-hyd-Dex NPs were generated by self-assembly in the pH neutral water solution with the assistance of PLGA; (4) HA was coated on the surface of Omi-hyd-Dex NPs through amido linkages and electrostatic interactions to produce Omi-hyd-Dex@HA NPs.

**Fig. 1:**
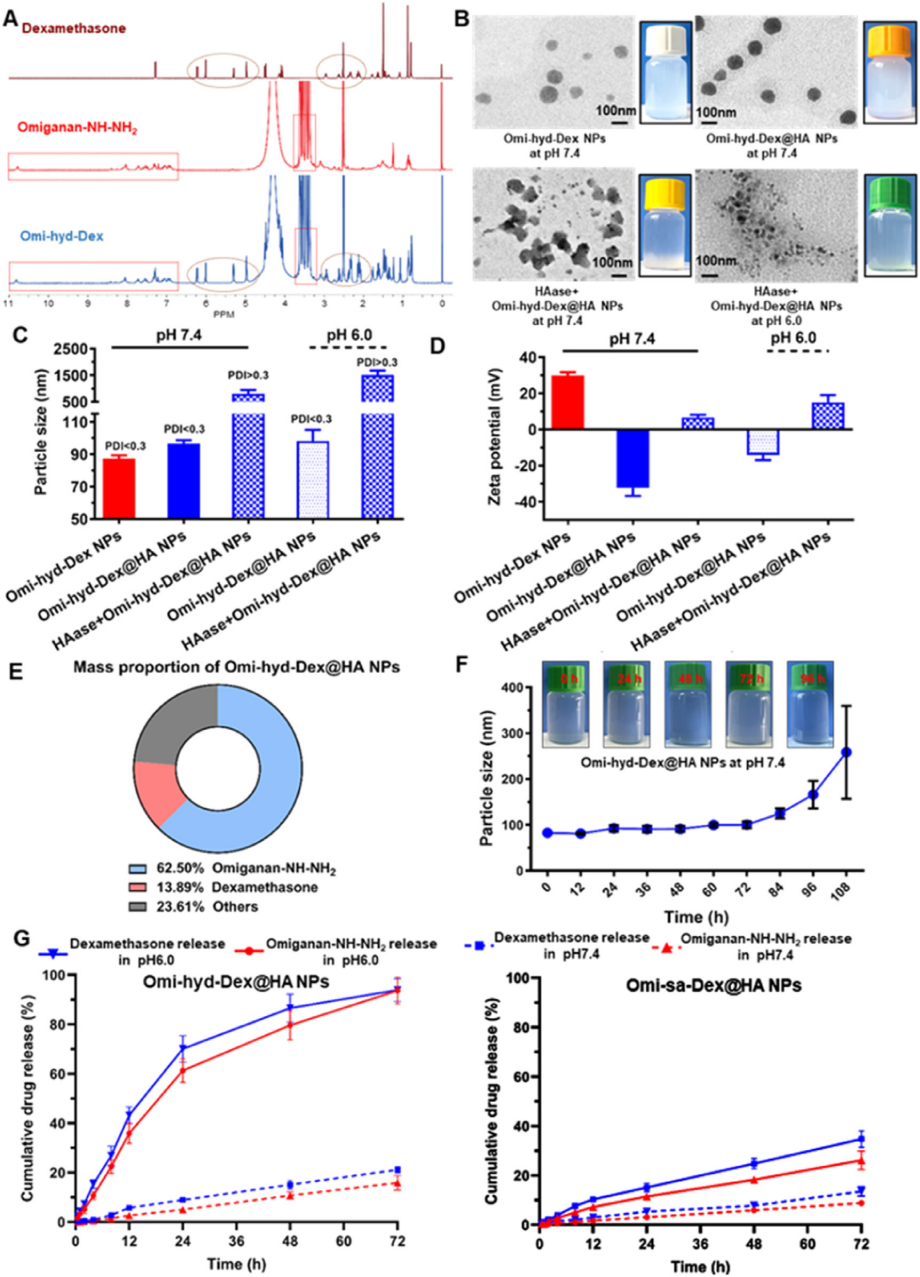
Preparation and characterization of Omi-hyd-Dex@HA NPs. (A) ^1^H NMR spectra of Dex, Omiganan-NH—NH_2_ and Omi-hyd-Dex in DMSO‑*d*_6_. (B) TEM images (scale bar 100 nm) and photographs of Omi-hyd-Dex NPs and Omi-hyd-Dex@HA NPs in different conditions. NPs (0.3 mg/ml) were suspended in buffers (pH 7.4 and 6.0) with or without HAase (150 U/ml) for 2 h at 37 °C. (C) Particle size (hydrodynamic diameter) of NPs (0.05 mg/ml) in buffer (pH 7.4 and 6.0) measured by DLS and their (D) surface zeta potential. (E) Mass proportion of Omi-hyd-Dex@HA NPs. (F) Particle size changes of Omi-hyd-Dex@HA NPs (0.05 mg/ml) during 108-h storage and the representative images of Omi-hyd-Dex@HA NPs at certain time points. (G) *In vitro* release profiles of Dex and Omiganan-NH—NH_2_ from Omi-hyd-Dex@HA NPs or Omi-sa-Dex@HA NPs in PBS (pH 7.4 or 6.0).

The ^1^HNMR spectrum of the Omi-hyd-Dex displayed both the characteristic peaks of Dex (5.00–6.25 ppm) and omiganan (3.0–4.0 ppm and 6.75–8.50 ppm), and mass spectrometric data confirmed the successful conjugation (Fig. 1A and **S1**). Due to HA coating on the Omi-hyd-Dex NPs cores, the diameter of the NPs measured with dynamic light scattering (DLS) increased from 87.3 nm to 96.6 nm (Figs. 1C and **S3, S4**). Moreover, the zeta potential of nanoparticles reversed from +29.8 mV to –32.7 mV after HA coating, corresponding to the carboxyl groups on the HA shell and the shielding of guanidine and amino groups on core-NPs (Fig. 1D and S4). By HPLC-MS analysis of NPs, the loading content of Omiganan-NH—NH_2_ and Dex in Omi-hyd-Dex@HA NPs were examined to be 62.5 and 13.89%, respectively (Fig. 1E). To determine the physiological stability, Omi-hyd-Dex@HA NPs were suspended in PBS (20% FBS) for 108 h. The hydrodynamic size changed little within 72 h, and gradually increased to 257 nm after 108 h, suggesting that the Omi-hyd-Dex@HA NPs could keep stable for 3 d in the physiological condition (Fig. 1F). Additionally, the results obtained from Fig. S5 further support these findings, as they demonstrate the physical and chemical stability of Omi-hyd-Dex@HA NPs in DMEM (40% mouse serum).

To precisely study the responsiveness of Omi-hyd-Dex@HA NPs, Omi-sa-Dex@HA NPs (the pH-responsive hydrazone bond was substituted by succinimide linkage) and Omi-hyd-Dex@PAA NPs (the HA coating was replaced by PAA) were fabricated and employed as the control NPs (**Figs. S2** and **S4**).

### 3.2 HAase- and pH-responsive behaviors of Omi-hyd-Dex@HA NPs

The HA coating on NPs not only served as the targeting ligand but also acted as a responsive framework for particle accumulation in IMEs. Many bacteria produced HAase, which could be the initiator of HA coating degradation and particle aggregation of Omi-hyd-Dex@HA NPs [45,46]. Moreover, the overproduced ROS and RNS in IMEs could also degrade the HA molecules in Omi-hyd-Dex@HA NPs [41,45]. To study the responsiveness of Omi-hyd-Dex@HA NPs, the nanoparticles were incubated with exogenous HAase for 2 h at 37 °C to simulate the effects of abundant HAase in IMEs. Notably, HAase treatment markedly changed the zeta-potential of Omi-hyd-Dex@HA NPs (from −14.2 mV to +15.1 mV at pH 6.0; from −32.3 mV to +7.3 mV at pH 7.4) and induced aggregation (diameter increased from 98.2 to 1585 nm at pH 6.0; from 96.6 to 807 nm at pH 7.4), indicating the HA degradation (Fig. 1B–1D). In contrast, Omi-hyd-Dex@HA NPs incubated in the buffer without HAase hardly changed in size or zeta potential, and the TEM images of NPs also confirmed these results (Fig. 1B).

To realize IMEs specific drug release, the hydrazone bond was used to connect the peptide and Dex, which would be cleaved in the acidic IMEs (pH 5.5–6.0) and lead to the release of Omiganan-NH—NH_2_ and Dex [47]. To study the acidity-responsive drug release behavior of Omi-hyd-Dex@HA NPs, phosphate buffers in different pH conditions (pH 7.4 or 6.0) were prepared to mimic blood circulation and inflammatory tissue microenvironment. In the neutral conditions (pH 7.4), the drug release rates of Omi-hyd-Dex@HA NPs and Omi-sa-Dex@HA NPs were both less than 21% within 72 h (Fig. 1G). In contrast, the release of both omiganan and Dex from Omi-hyd-Dex@HA NPs was dramatically accelerated in the acidic condition. Whereas, the drug release profiles of Omi-sa-Dex@HA NPs hardly changed at pH 6.0. These results suggest that the hydrazone linkage between omiganan peptide and Dex endowed the Omi-sa-Dex@HA NPs with pH-responsive drug release properties, which would specifically release them to suppress the infection and inflammation in IMEs.

### 3.3 Biocompatibility of Omi-hyd-Dex@HA NPs

**Fig. 2:**
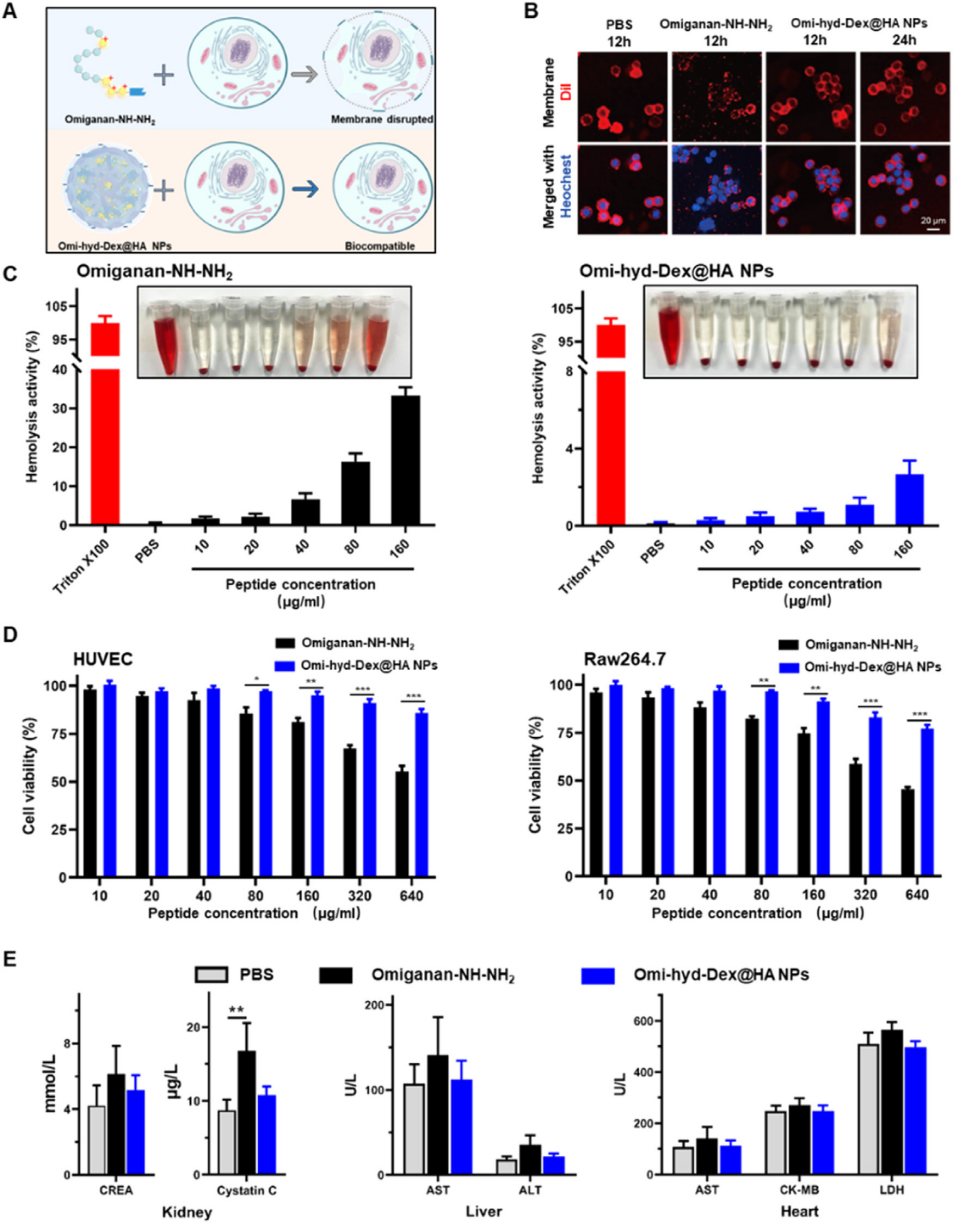
Biocompatibility of Omi-hyd-Dex@HA NPs *in vitro* and *in vivo*. (A) Schematic illustration of Omi-hyd-Dex@HA NPs avoiding the membrane disruption effect of Omiganan-NH—NH_2_. (B) Cell membrane staining with DiI (red) after Raw264.7 macrophage cells treated with PBS, Omiganan-NH—NH_2,_ or Omi-hyd-Dex@HA NPs for 12–24 h. The nuclei were stained with Hoechst 33342 (blue). (C) Hemolysis assay of Omiganan-NH—NH_2_ and Omi-hyd-Dex@HA NPs. (D) The cytotoxicity of Omi-hyd-Dex@HA NPs in HUVEC and Raw264.7 cells. (E) The sera from the healthy mice that received treatment were biochemically analyzed. The mice (*n* = 3) were intravenously injected with PBS, Omiganan-NH—NH_2_, or Omi-hyd-Dex@HA NPs (10 mg/kg, peptide equivalent; four times; at 2-d intervals) and blood was drawn the day after the last injection. CREA, creatinine; AST, aspartate aminotransferase; ALT, alanine aminotransferase; CK-MB, creatine kinase-MB; LDH, lactic acid dehydrogenase. Data are shown as mean ± SD, *P < 0.05, **P < 0.01, ***P < 0.001, N.S., not significant.

Despite the potent broad antimicrobial activity, most cationic AMPs including omiganan could damage mammalian cell membranes by disrupting the phospholipids' physiological structure, which limits their systemic clinical application [39]. Based on our design, we hypothesized the HA coating on the nanoformulation might shield the positive moieties to abrogate this side effect of omiganan, as shown in Fig. 2A. To test this hypothesis, we evaluated the cell membrane morphology of murine macrophage cells (Raw264.7) after exposure to Omiganan-NH—NH_2_ or Omi-hyd-Dex@HA NPs. The cell membrane was visualized using a red fluorescence agent (DiI). As shown in Fig. 2B, obvious distorted and discontinuous membranes were found on the cells treated with Omiganan-NH—NH_2_ for only 12 h. On the contrary, cell morphology remained intact in the cells exposed to Omi-hyd-Dex@HA NPs for 24 h, indicating that the membrane-disrupting effect of omiganan was abrogated by the rational design of Omi-hyd-Dex@HA NPs. Next, we evaluated the hemolysis rate and the cytotoxicity on healthy somatic cells. As shown in Fig. 2C, a dose-dependent tendency of hemolysis activity was observed in the Omiganan-NH—NH_2_ group, indicating the membrane damage effect of Omiganan-NH—NH_2_. As expected, Omiganan-NH—NH_2_ showed significant cytotoxicity to HUVECs and Raw264.7 cells at a concentration higher than 80 µg/ml (Fig. 2D). In contrast, Omi-hyd-Dex@HA NPs induced significantly lower hemolysis and cell death for both cell lines compared with Omiganan-NH—NH_2_. Even at a concentration equivalent to 640 µg/ml Omiganan-NH—NH_2_, the nanoformulation induced less than 20% of cell death, indicating good cytocompatibility of Omi-hyd-Dex@HA NPs.

According to existing clinical data, some AMPs such as polymyxin B could induce nephrotoxicity and neurotoxicity via intravenous administration [48]. By employing fluorescence detection with FITC-labeled NPs in serum, we observed that the majority of the drug would be eliminated within 24 h (Fig. S6). To evaluate the systemic toxicity of Omi-hyd-Dex@HA NPs upon multiple administrations, we subjected healthy mice to four doses of either free omiganan or Omi-hyd-Dex@HA NPs through intravenous injection into the tail (peptide equivalent of 10 mg/kg). One day after the final injection, we collected the blood sera for a medical biochemical examination. Slightly higher CREA and significantly higher blood cystatin C were detected in the Omiganan-NH—NH_2_ treated mice, indicating acute nephrotoxicity (Fig. 2E). By contrast, the mice treated with Omi-hyd-Dex@HA NPs showed little change in biochemical analysis. Meanwhile, the H&E staining of major organs from mice receiving Omi-hyd-Dex@HA NPs did not show any obvious change compared with the PBS group (Fig. S7), indicating the excellent biocompatibility of Omi-hyd-Dex@HA NPs *in vivo*.

### 3.4 Targeting and penetration effects of Omi-hyd-Dex@HA NPs

**Fig. 3:**
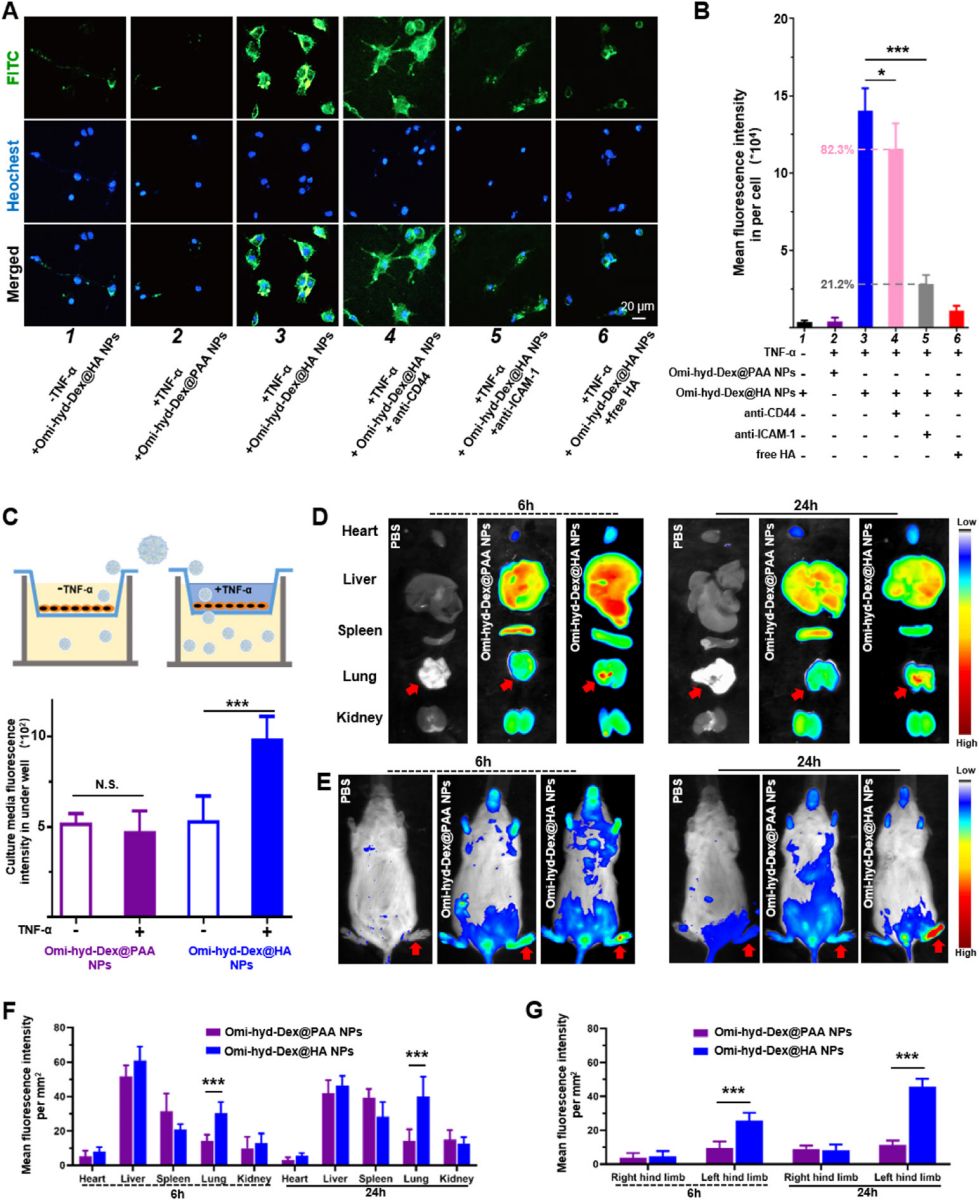
Omi-hyd-Dex@HA NPs targeting and penetrating inflamed endothelium cells by ICAM-1 recognition and accumulating in bacterial infectious tissues. (A) Confocal laser scanning microscope images showed the ICAM-1/HA-mediated interaction between NPs (FITC-labeled; green) and TNF-α-activated HUVECs. The nuclei were stained with Hoechst 33342 (blue). (1) Cells treated with Omi-hyd-Dex@HA NPs without TNF-α activation; (2) TNF-α-activated cells treated with Omi-hyd-Dex@PAA NPs; (3) TNF-α-activated cells treated with Omi-hyd-Dex@HA NPs; (4) TNF-α-activated cells treated with Omi-hyd-Dex@HA NPs after CD44 blocking-up; (5) TNF-α-activated cells treated with Omi-hyd-Dex@HA NPs after ICAM-1 blocking-up; (6) TNF-α-activated cells treated with Omi-hyd-Dex@HA NPs after free HA competitive inhibition. (B) Mean FITC fluorescence intensity in each group of HUVECs, which was calculated by adding the total FITC fluorescence intensity in each image together and dividing by the number of Hoechst 33342 labeled cells. (C) Omi-hyd-Dex@HA NPs penetrated each well of HUVECs after 24-h incubation. (D) Omi-hyd-Dex@HA NPs accumulated in *K.P.-*infected (tracheal injection) lung tissue. One dose of PBS or NPs (5 mg/kg; DiR-labeled) was administrated by i.v. injection to *K.P.-*induced pneumonia model mice (*n* = 3), and the major organs were taken out for IVIS imaging after 6 or 24 h. (E) Omi-hyd-Dex@HA NPs accumulated in *S. aureus-*infected footpad (left hind limb). One dose of PBS or NPs (5 mg/kg; DiR-labeled) was administrated by i.v. injection on *S. aureus-*induced footpad infection model mice (*n* = 3) and mice were imaged by IVIS after 6 or 24 h. (F) The mean fluorescence intensity per mm^2^ in major organs after i.v. injection for 6 or 24 h. *K.P.-*infected tissue: lung. (G) The mean fluorescence intensity per mm^2^ in hind limbs 6 or 24 h after i.v. injection. *S. aureus*-infected tissue: left hind limb. Data are shown as mean ± SD, *P < 0.05, ***P < 0.001, N.S., not significant.

To determine whether Omi-hyd-Dex@HA NPs could specifically target inflamed tissue cells, we prepared FITC-labeled Omi-hyd-Dex@HA NPs and Omi-hyd-Dex@PAA NPs for fluorescence microscopy experiments. We chose TNF-α-activated HUVECs to simulate the inflamed endothelium. As shown in Fig. 3A and 3B, few Omi-hyd-Dex@HA NPs were observed in the untreated HUVECs. In the TNF-α-activated HUVECs, a remarkably higher amount of Omi-hyd-Dex@HA NPs were internalized in the cells. However, much fewer Omi-hyd-Dex@PAA NPs were observed in the TNF-α-activated HUVECs compared with Omi-hyd-Dex@HA NPs due to PAA coating instead of HA. Notably, this difference was suppressed by the competitive inhibition of free HA molecules, indicating the HA-mediated endocytosis interaction. Previous studies reported that the inflammation-targeting property of HA was mainly dependent on CD44 and ICAM-1 receptor recognition [41]. Hence, we prepared anti-CD44 and anti-ICAM-1 blocked HUVECs by monoclonal antibody inhibition method for further research. We found a remarkably lower FITC fluorescence intensity after anti-ICAM-1 blockage (> 70% decrease) than anti-CD44 (< 20% decrease). These results indicated that ICAM-1 receptor might be the main target in inflamed endothelial cells for HA-coated NPs.

Further, to investigate whether NPs could across the inflamed endothelial cells to penetrate the IMEs, we prepared a monolayer of HUVECs to simulate blood vessel endothelium by Transwell culture plates. As shown in Fig. 3C and S8, only a slight amount of Omi-hyd-Dex@PAA NPs penetrated through the cell layer with or without TNF-α activation to the lower chamber. In contrast, Omi-hyd-Dex@HA NPs transportation was significantly enhanced by TNF-α activation and blocked by anti-ICAM-1, hinting that the HA/ICAM-1-mediated recognition process in the IMEs promoted the targeting and penetration of Omi-hyd-Dex@HA NPs.

### 3.5 Omi-hyd-Dex@HA NPs specifically accumulated in the bacteria-infected lung tissue and footpad

A variety of infections could occur in almost all organs and tissues, which might set barriers to drug delivery. However, the inflamed vascular endothelial with disrupted structure and increased permeability in IMEs might facilitate NPs accumulation through the EPR effect. Besides, HA/ICAM-1-mediated targeting and penetration effects might further promote Omi-hyd-Dex@HA NPs accumulation into IMEs. To simulate these complex scenarios, we established *K.P.* (G-) induced pneumonia model and *S. aureus* (G+) induced footpad infection model for assessing the Omi-hyd-Dex@HA NPs biodistribution *in vivo*, respectively.

Pneumonia mouse models were established through intratracheal administration of *K.P.* and a single dose of NPs (DiR-labeled) was intravenously injected 24 h later. After 6 or 24 h, mice were sacrificed and the fluorescence intensity in the main organs was investigated. As shown in Fig. 3D–3G, Omi-hyd-Dex@HA NPs and Omi-hyd-Dex@PAA NPs showed similar bio-distribution features, in which NPs mainly distributed in the liver, lung, and spleen at 6 to 24 h after injection. Notably, Omi-hyd-Dex@HA NPs-treated mice showed much stronger DiR fluorescence intensity in lung tissues compared with the Omi-hyd-Dex@PAA NPs group (∼2-fold) at 6 h. Moreover, more Omi-hyd-Dex@HA NPs accumulated in the lungs at 24 h. Similarly, in *S. aureus*-induced footpad infection model, the DiR-labeled Omi-hyd-Dex@HA NPs specifically accumulated in the infected foot but not in the healthy ones, as stronger fluorescence of DiR was observed in the *S. aureus*-infected foot compared with the uninfected ones, and was significantly higher than that of Omi-hyd-Dex@PAA NPs-treated mice. These results indicate that NPs might passively accumulate into infected tissues by the EPR effect, while Omi-hyd-Dex@HA NPs showed significantly more NPs accumulation via HA/ICAM-1-mediated targeting ability.

Throughout the above results, we achieved the specific targeting of ICAM-1 receptor at the inflammatory site by bio-friendly encapsulation of NPs core with HA. Additionally, after NPs entered the microvessels around IMEs from the blood circulation, HAase in the microenvironment would degrade the HA coating to reverse the surface charge of NPs, which in turn promotes the precipitation and retention of NPs in the ruptured tissue of the infection site, further enhancing the targeting delivery efficiency of NPs. Our rational design ensures that the HA coating on NPs can successfully carry out its functions in a sequential manner. By this process, the multiple biological functions of HA are fully utilized.

### 3.6 Antimicrobial activity of Omi-hyd-Dex@HA NPs in vitro

To verify whether Omi-hyd-Dex@HA NPs inherited the antimicrobial activity of omiganan, the MICs were tested by the standardized agar doubling-dilution method as shown in Table 1. Omi-hyd-Dex@HA NPs and free omiganan showed similar extended broad-spectrum antibacterial activity for G+ bacteria, G- bacteria and fungi pathogens, including drug-sensitive strains (*S. aureus, P. aeruginosa, K.P., Candida albicans*) and drug-resistant strains (MRSA, MDR-*K.P.*). The MICs of Omi-hyd-Dex@HA NPs showed none or only a one-fold increase compared with the free Omiganan-NH—NH_2_ group. In addition, free Dex did not show any antimicrobial activity for the tested pathogens, indicating that Dex moieties of Omi-hyd-Dex@HA NPs did not contribute to the bacterial killing effect. In contrast, vancomycin, as a standard drug regimen for drug-resistant G+ strains, showed inefficacious for G- strains and fungus. Similar results were also observed in oxacillin-treated groups. Therefore, Omi-hyd-Dex@HA NPs retained the broad-spectrum antimicrobial activity of omiganan.

**Table 1:** MICs of Omi-hyd-Dex@HA NPs, Omiganan-NH—NH_2_ and reference antibiotics against different types of pathogens (µg/ml, as omiganan equivalent).

**Common pathogens in the clinic**		**Omi-hyd-Dex@HA NPs**	**Omiganan-NH—NH_2_**	**Dex**	**Oxacillin**	**Vancomycin**
**Strains/Classification**						
***S. aureus* (ATCC 29,213)**	***G*+**	**8**	**4**	**>64**	**0.5**	**<0.5**
**MRSA**		**16**	**8**	**>64**	**16**	**4**
***P. aeruginosa***	**G-**	**32**	**16**	**>64**	**32**	**>64**
***K.P.***		**16**	**16**	**>64**	**16**	**64**
**MDR-*K.P.***		**16**	**8**	**>64**	**>64**	**>64**
***Candida albicans***	**Fungus**	**32**	**16**	**>64**	**>64**	**>64**

### 3.7 Omi-hyd-Dex@HA NPs killed bacteria rapidly through membrane disruption

**Fig. 4:**
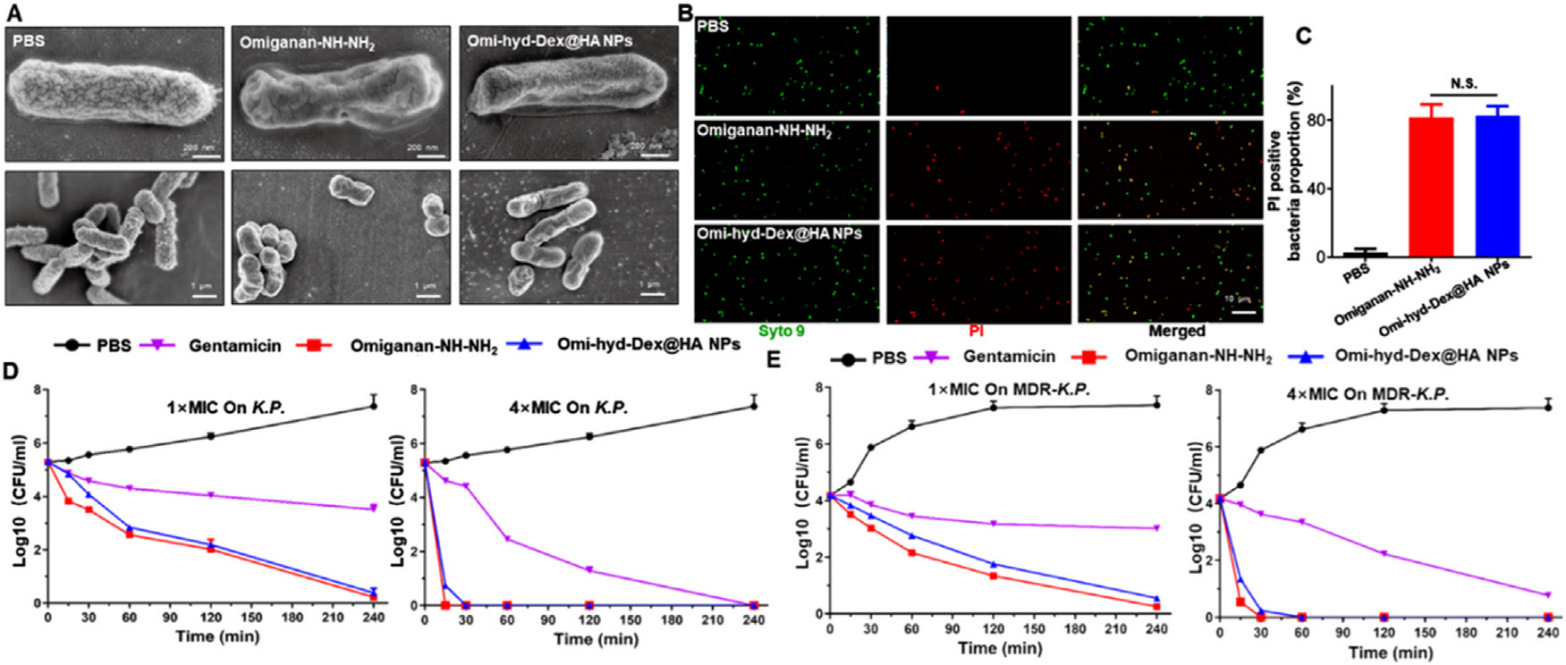
Omi-hyd-Dex@HA NPs retained the antibacterial activity of Omiganan-NH—NH_2_. (A) Representative SEM images of *K.P.* after treatment with PBS, Omiganan-NH—NH_2_ or Omi-hyd-Dex@HA NPs (2× MIC) for 4 h. (B-C) LIVE/DEAD stained *K.P.* under confocal laser scanning microscope. Red for PI-stained nucleoplasm, green for SYTO 9-stained bacterial membrane. Bacteria were treated with PBS, omiganan, or Omi-hyd-Dex@HA NPs (2× MIC) for 1 h, followed by staining with SYTO 9 (live and dead bacteria) and PI (dead bacteria). PI-positive bacteria were counted. N.S., not significant. (D-E) Time-kill kinetics assay of Omi-hyd-Dex@HA NPs, omiganan, or Gentamicin against *K.P.* and MDR-*K.P.* at different concentrations. Gentamicin was used as a positive control.

Like most cationic AMPs, omiganan can rapidly increase cytoplasmic membrane permeability, leading to fast bacterial clearance [29]. The membrane disruption effect of Omi-hyd-Dex@HA NPs was observed by SEM. As shown in Fig. 4A, both Omi-hyd-Dex@HA NPs and omiganan caused severe membrane shrinking and obvious morphological changes within 4 h on *K.P.* Combined with confocal results, Omi-hyd-Dex@HA NPs and omiganan showed similar PI-stained proportion (Fig. 4B and 4C), indicating that both omiganan and Omi-hyd-Dex@HA NPs markedly increased the permeability of bacteria membranes.

Time-kill kinetics were investigated by CFU assay to examine whether the fast-sterilization ability of omiganan was hampered by NPs formulation. Gentamicin, as a *K.P.*-sensitive antibiotic, was employed for positive control. As shown in Fig. 4D-4E, at 1× MICs, both Omi-hyd-Dex@HA NPs and omiganan almost killed all the *K.P.* and the drug-resistant counterpart (MDR-*K.P.*) within 4 h. Further, at 4× MICs, both Omi-hyd-Dex@HA NPs and omiganan effectively killed all bacteria in less than 1 h. For both strains of drug-sensitive and -resistant *K.P.,* Omi-hyd-Dex@HA NPs exhibited good antibacterial activity. In contrast, Gentamicin required a much longer time and higher drug concentration to fully eradicate bacteria (more than 4 h). Combined with MIC testing results, Omi-hyd-Dex@HA NPs exhibited much faster kill kinetics than the conventional antibiotics, and showed potential to mitigate bacterial resistance.

### 3.8 Anti-inflammatory effects of Omi-hyd-Dex@HA NPs in vitro

**Fig. 5:**
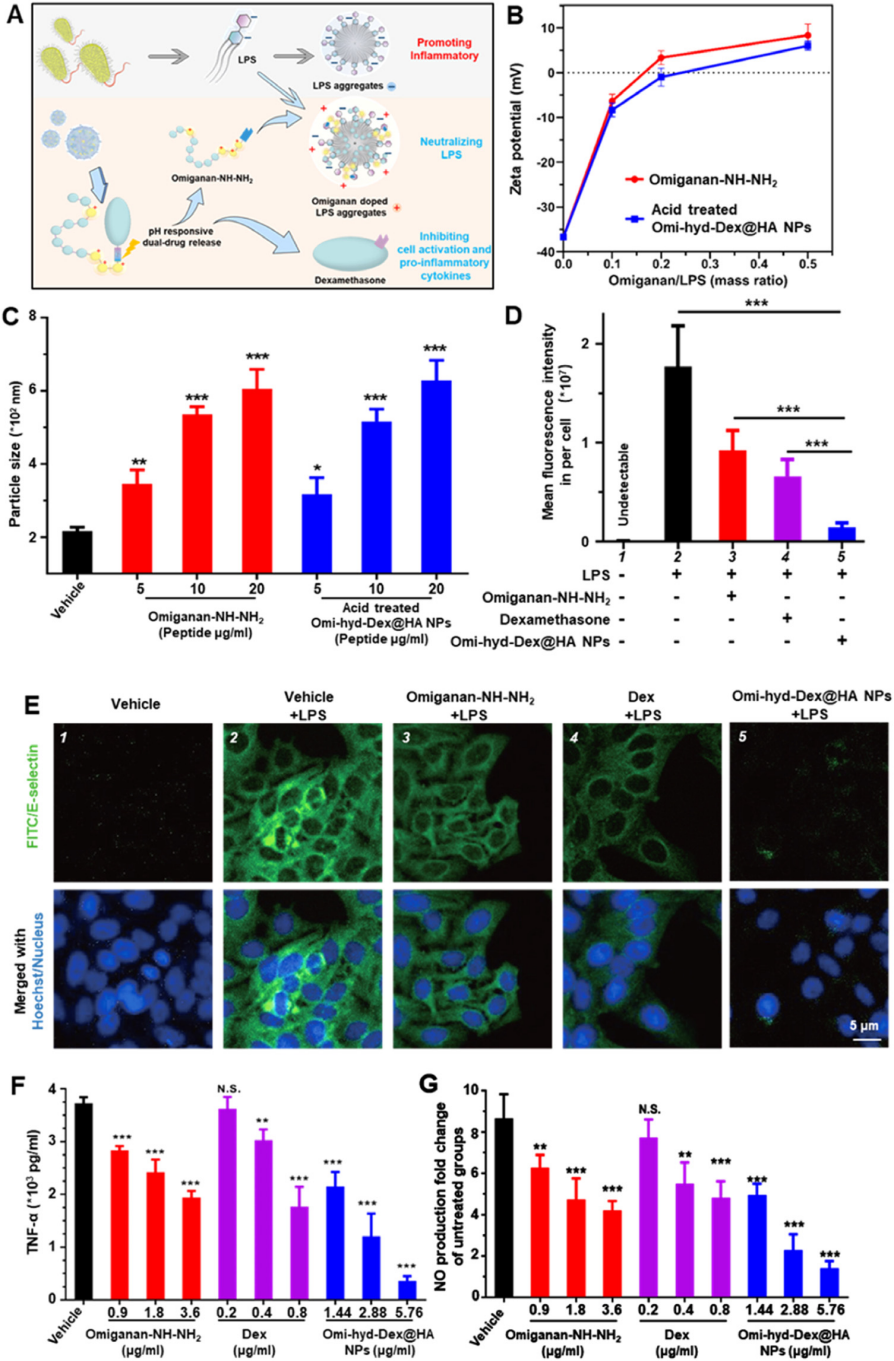
The LPS neutralization and dual anti-inflammatory activity of Omi-hyd-Dex@HA NPs *in vitro*. (A) Schematic illustration of the dual anti-inflammatory function of Omi-hyd-Dex@HA NPs. (B-C) The released omiganan from Omi-hyd-Dex@HA NPs altered the particle state of LPS aggregation. The particle size and surface zeta potential were determined by the Malvern DLS device with an electrode probe. (D-E) E-selectin expression in mouse primary microvascular endothelial cells. LPS (100 ng/ml) with Omi-hyd-Dex@HA NPs or free drugs were mixed in DMSO solution and added into cell culture media for 24 h. (Omi-hyd-Dex@HA NPs 10 µg/ml, equivalent to Omiganan-NH—NH_2_ 6.25 µg/ml or Dex 1.389 µg/ml). Vehicle (DMSO)-treated cells were employed as a negative control. (F) TNF-α and (G) NO production by LPS-challenged RAW264.7 macrophages after administration of Omi-hyd-Dex@HA NPs, Dex or Omiganan-NH—NH_2_. Data are shown as mean ± SD, *P < 0.05, **P < 0.01, ***P < 0.001, N.S., not significant. (1.44 mg of Omi-hyd-Dex@HA NPs contains 0.9 mg of Omiganan-NH—NH_2_ and 0.2 mg of Dex; LPS 100 ng/ml).

The pro-inflammation activity of LPS is dependent on its aggregation state, which can be interfered with by most cationic AMPs [33,^34^,^37^,49]. Thanks to their amphipathic and cationic features, cathelicidin family AMPs have been reported to possess great LPS neutralization activity [34]. To test whether Omi-hyd-Dex@HA NPs could modulate the structure and surface charge of LPS aggregates, we prepared LPS aggregates from *E. coli*. O111:B4 for detection. As shown in Fig. 5B-5C and **S9**, the vehicle (45 µl DMSO added into 2 ml LPS aggregates) did not affect the hydrodynamic size (from 218.7 to 216.6 nm) and zeta potential (from −36.33 to −36.57 mV) of LPS aggregates. However, the addition of free Omiganan-NH—NH_2_ or acid-pretreated Omi-hyd-Dex@HA NPs induced similar negative charge neutralization and charge reversal (from −6.45 to +8.65 mV; from −8.73 to +6.11 mV). In the meantime, the diameters of LPS aggregates increased in a dose-dependent profile (from 344 to 607 nm; from 316 to 622 nm). These results suggested that the degraded derivatives of acid-pretreated Omi-hyd-Dex@HA NPs could change the structure and surface charge of LPS aggregates in the same way as free Omiganan-NH—NH_2_, which might intervene in the pro-inflammation activity of LPS.

IMEs-released LPS can not only initiate adjacent endothelial cell activation for immune cell recruitment but also potently motivate immune cells to responsively produce proinflammatory substances like TNF-α and NO which trigger further inflammatory cascades [50]. In an inflamed state, E-selectin is one of the main immune cell adhesion molecules expressed on endothelial cells for neutrophil recruitment, which might amplify inflammation [51]. To assess whether Omi-hyd-Dex@HA NPs could reverse LPS-induced endothelial cell activation, we prepared LPS-challenged mouse primary microvascular endothelial cells for visualizing E-selectin expression by immunofluorescence. After co-culture with LPS for 2 h, mouse primary microvascular endothelial cells were treated with NPs, free Dex, or Omiganan-NH—NH_2_ alone for 12 h, respectively. As shown in Fig. 5D and 5E, we found both free Dex and omiganan could down-regulate the LPS-caused E-selectin overexpression. Notably, Omi-hyd-Dex@HA NPs showed comprehensive blocking ability indicated by the slightest green fluorescence of E-selectin. To test whether Omi-hyd-Dex@HA NPs could inhibit the pro-inflammation effect of LPS, free Omiganan-NH—NH_2_, Dex or Omi-hyd-Dex@HA NPs were used to treat the LPS-challenged Raw264.7 cells for 24 h. Consistent with the LPS aggregates interference results, the addition of free Omiganan-NH—NH_2_ or Omi-hyd-Dex@HA NPs made LPS-challenged cells produce relatively less TNF-α and NO, and the decrease was positively correlated with drug concentration, as shown in Fig. 5F and 5G. Compared to free Dex or Omiganan-NH—NH_2_, the Omi-hyd-Dex@HA NPs showed much stronger anti-inflammation activity. Overall, the integrated LPS neutralizer Omiganan-NH—NH_2_ and anti-inflammatory Dex together secured the potent inflammation inhibitory mechanism of Omi-hyd-Dex@HA NPs (Fig. 5A).

### 3.9 Omi-hyd-Dex@HA NPs blocked systemic inflammatory response in LPS-induced sepsis mice

**Fig. 6:**
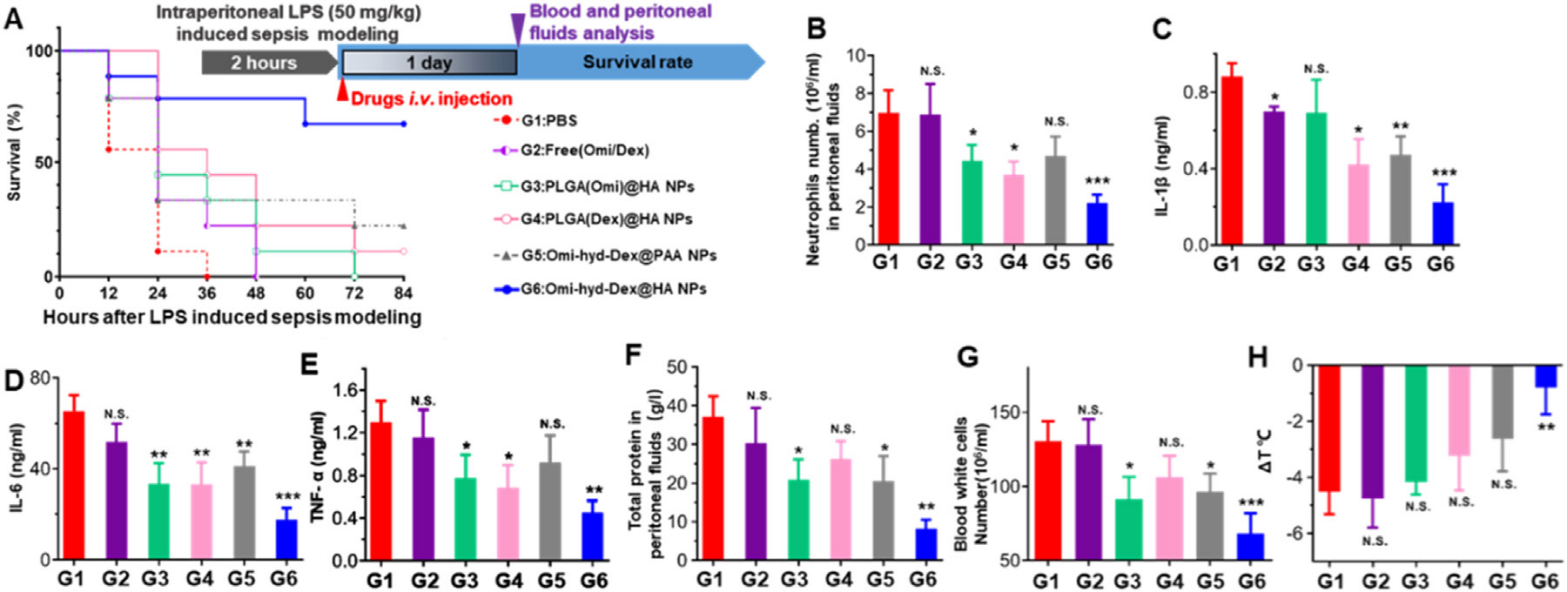
The comprehensive anti-inflammation therapeutic efficacy of Omi-hyd-Dex@HA NPs *in vivo*. (A) Experiment timeline and survival percentages (*n* = 9) of lethal dose LPS-induced sepsis model mice receiving treatments. Sepsis mice treated with PBS were used as a negative control. (B) Neutrophils number, (C-E) pro-inflammatory cytokines concentrations, and (F) total protein mass quantification in peritoneal fluid collected from sepsis mice receiving various treatments (*n* = 3). (G-H) Blood white cells number and body temperature change 1 d after treatment. Compared with body temperature before modeling. Data are shown as mean ± SD, *P < 0.05, **P < 0.01, ***P < 0.001, N.S., not significant.

To test whether Omi-hyd-Dex@HA NPs could inhibit the pro-inflammation effect of LPS *in vivo*, a lethal dose of LPS was intraperitoneally injected into mice mimicking the initial stage of sepsis, which caused SIRS and led to death in 48 h. As shown in Fig. 6A, after a single dose of Omi-hyd-Dex@HA NPs by i.v. administration, 88.8% (8/9) of mice survived for 84 h. However, the Omi-hyd-Dex@PAA NPs only resulted in 55.5% (5/9) survival, which might be ascribed to the absence of HA/ICAM-1 receptor-mediated IMEs targeting. In contrast, the Dex or peptide-loaded NPs [PLGA(Omi)@HA NPs and PLGA(Dex)@HA NPs)] or free drug combination (Omi/Dex) demonstrated very limited protection effects with less than 20% mice survival.

For assessing the inflammation level in the peritoneal cavity, peritoneal fluid was collected by PBS intraperitoneal injection and extraction 1 d after treatment. Neutrophil activation and infiltration in IMEs signaled the initial period of massive amplification of the inflammatory response [14]. We found a marked decrease in infiltrated neutrophils and pro-inflammatory cytokines production (TNF-α, IL-6, IL-1β) after Omi-hyd-Dex@HA NPs treatment as shown in Fig. 6B–6E. Further, the total protein content also decreased after Omi-hyd-Dex@HA NPs treatment in accordance with the reduced neutrophil infiltration, suggesting vascular integrity in the peritoneal cavity (Fig. 6F). All these results revealed that Omi-hyd-Dex@HA NPs blocked the primary lesions (in the peritoneal cavity) of LPS-induced inflammation effectively.

SIRS, as a complication of LPS-induced sepsis, is an exaggerated systematic immunity defense response, which will cause dysregulated cytokine storms and septic death [16,52]. SIRS is characterized by sustained abnormal body temperature and rapid amplification of peripheral blood leucocytes [52]. Thus, the mice's body temperature and white blood cells were also monitored 1 d after treatment. The decrease of body temperature and blood leucocytes was significantly weakened by Omi-hyd-Dex@HA NPs, while other treatments were insufficient to suppress temperature heightening and leucocytes infiltration, indicating that the SIRS was effectively controlled by Omi-hyd-Dex@HA NPs (Fig. 6G and H). Taking into account the level of inflammation in the peritoneal cavity and the comparison of data with healthy mice (Fig. S10), these results suggested that Omi-hyd-Dex@HA NPs avoided SIRS progression by inhibiting regional inflammation at the initial period of sepsis.

### 3.10 Omi-hyd-Dex@HA NPs reduced CLP-induced polymicrobial sepsis mortality and pro-inflammatory cytokines production with lowered DIC possibility

**Fig. 7:**
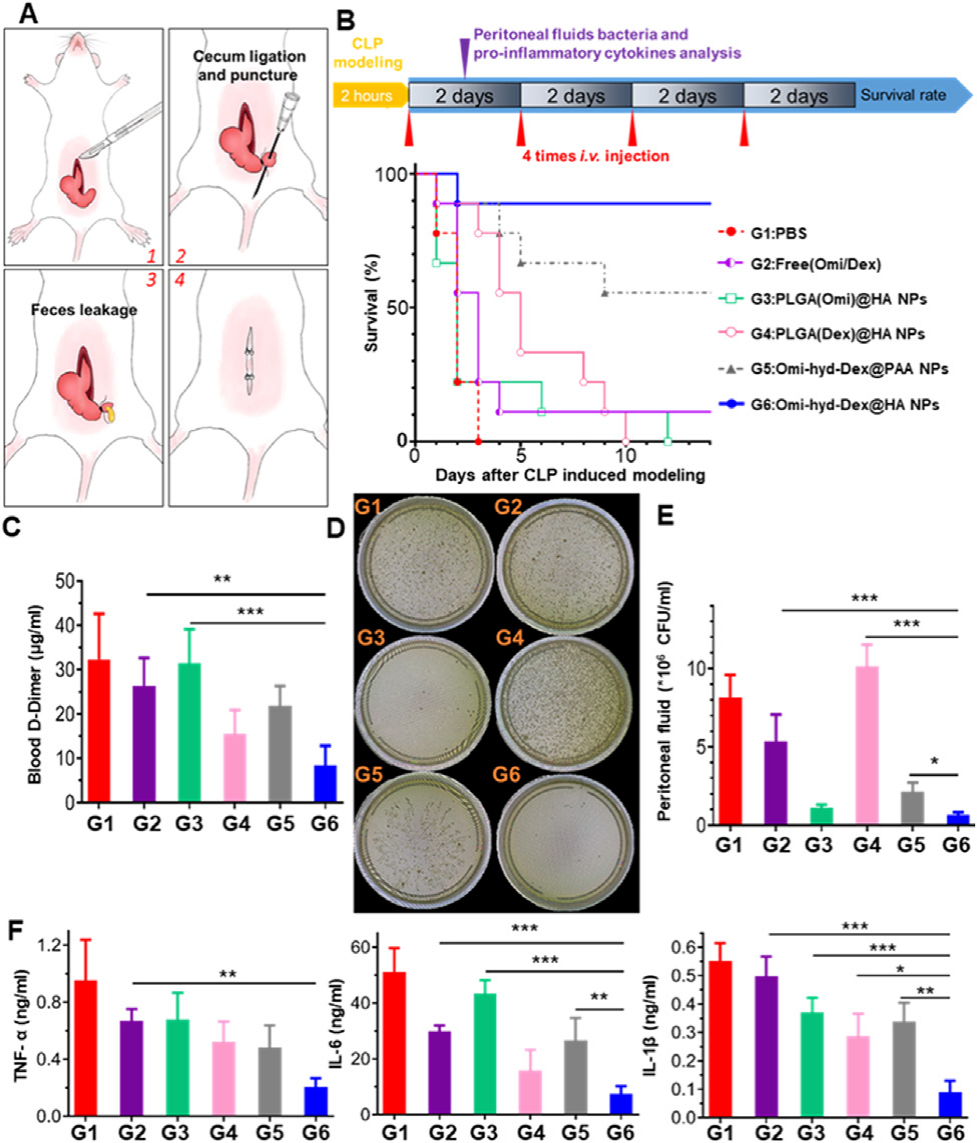
Rounds of Omi-hyd-Dex@HA NPs treatment effectively controlled CLP-induced polymicrobial sepsis and reduced bacterial proliferation, d-Dimer level, and pro-inflammatory cytokines production. (A) Schematics of CLP modeling process. About 30% of the full length of the cecum was ligated and punctured with a 26 G needle for feces leakage. (B) Experiment timeline and survival percentages (*n* = 9) of CLP model mice receiving treatments. (C) Blood serum d-Dimer detection (*n* = 3) on Day 1 after treatment. (D) Representative photograph and (E) bacterial colonies number of peritoneal fluid inoculated on BHI agar plates in aerobic condition for 18 h. (F) Pro-inflammatory cytokines concentrations (*n* = 3) in peritoneal fluid of the mice receiving treatment. Data are shown as mean ± SD, *P < 0.05, **P < 0.01, ***P < 0.001, N.S., not significant.

Despite the strong anti-inflammation ability of Omi-hyd-Dex@HA NPs in the LPS-induced sepsis model, these mice only received one round of treatment, which was different from the clinical treatment mode and did not involve pathogens. CLP-induced polymicrobial sepsis model was constructed as shown in Fig. 7A and S11. It is the golden standard model for sepsis research because it resembles the typical progression and characteristics of clinical septic patients [27,53]. In addition, four rounds of i.v. treatment (2 d intervals; 5 mg/kg omiganan or 1.1 mg/kg Dex equivalently) were operated to mimic the clinic therapy regimen. In the PBS-treated group, all mice died within 3 d HA-coated single drug-loaded NPs [PLGA(Omi)@HA NPs and PLGA(Dex)@HA NPs] showed 11.1% (1/9) survival in 14 d Omi-hyd-Dex@PAA NPs saved 55.6% (5/9) mice in 14 d In sharp contrast, Omi-hyd-Dex@HA NPs sufficiently saved the CLP mice with a survival rate of up to 88.9% (8/9), as shown in Fig. 7B.

Recognized as the deadliest complication in sepsis, DIC has a strong positive correlation with the d-Dimer and FDP level [17,54]. As shown in Fig. 7C and S12, the Omi-hyd-Dex@HA NPs group showed the lowest d-Dimer and FDP level, indicating the lowest DIC possibility. For assessing the microbial density in the feces-contaminated peritoneal cavity, bacterial culture for colony analysis with or without oxygen on BHI agar plates was conducted. As shown in Fig. 7D, 7E and S14, both the density of aerobic bacteria and anaerobic bacteria reduced dramatically just after one dose of Omi-hyd-Dex@HA NPs injection. Simultaneously, the production of pro-inflammatory cytokines (TNF-α, IL-6, IL-1β) reduced significantly. Overall, multi-rounds of Omi-hyd-Dex@HA NPs injection, simulating a clinical regimen, consistently exhibited the most effective therapeutic effect among all the aforementioned treatment groups. Furthermore, the data from this group closely resembled that of the sham surgery group (Fig. S13), suggesting that this treatment approach achieved outcomes similar to those in the absence of sepsis. These ideal results are likely due to the following factors: (1) the HA coating-mediated NPs targeting and accumulation into IMEs, (2) the potent antibacterial activity of omiganan moieties in NPs, and (3) the comprehensive inflammation-blocking abilities of omiganan and Dex.

### 3.11 Omi-hyd-Dex@HA NPs decreased sepsis mortality caused by clinically isolated pathogenic bacteria

**Fig. 8:**
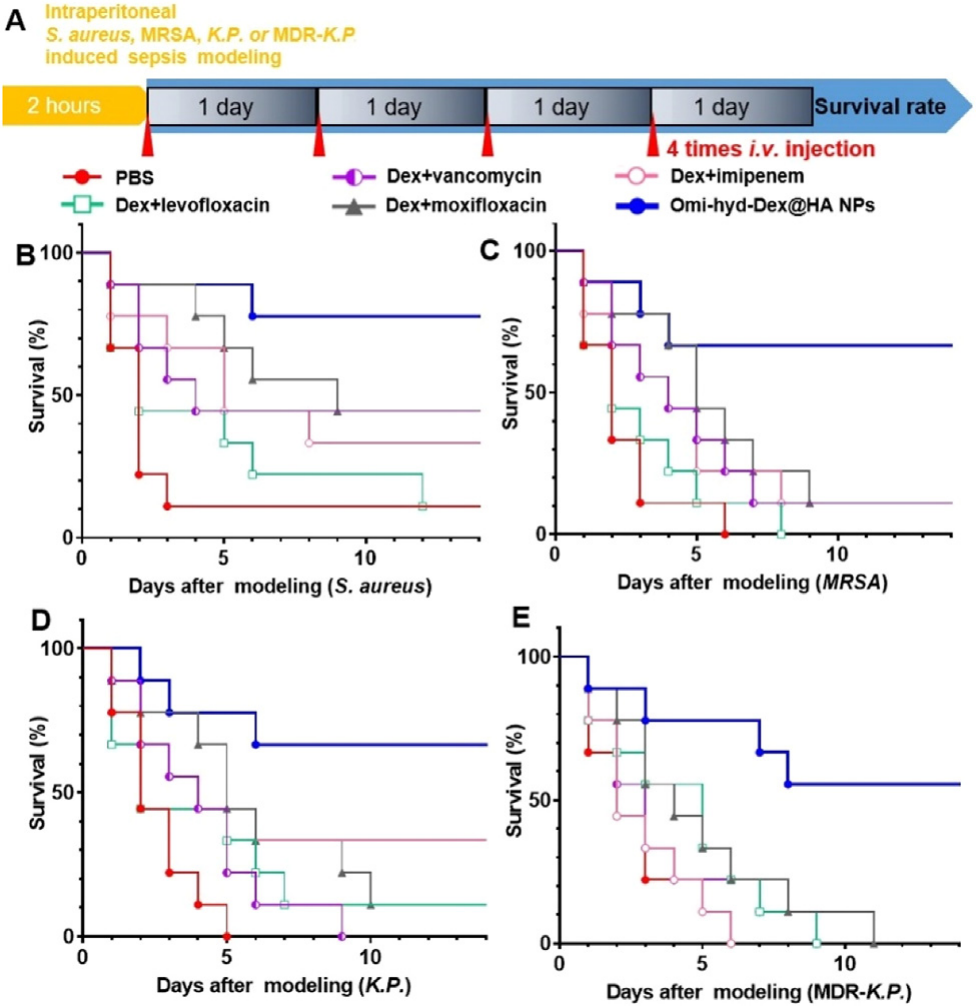
Simulation of Omi-hyd-Dex@HA NPs-based emergency therapeutic regimen against sepsis induced by four kinds of clinically isolated pathogenic bacteria. (A) Experiment timeline and legend name of each group. Mice were given intraperitoneal injections of four kinds of clinically isolated pathogenic bacteria for sepsis modeling. Mice with sepsis treated with PBS were used as a negative control. Drugs were administered once 1 d by i.v. injection for 4 times after modeling. (B-E) Survival percentages (*n* = 9 per group) of different bacteria-induced sepsis mice receiving treatments within 14 d

Based on the guidelines of Surviving Sepsis Campaign 2021, antimicrobials should be quickly administered within 1 h after confirmed diagnosis [4,55]. For the absence of antimicrobial susceptibility results in an emergency, it is unavoidable to apply conventional empirical therapy, which might be ineffective for rapid source control and even cause drug resistance [33]. To test the efficacy of Omi-hyd-Dex@HA NPs in treating sepsis caused by various pathogenic bacteria in the clinic, we prepared four kinds of sepsis mouse models by intraperitoneal injection of *S. aureus* (G+) and *K.P*. (G-) as well as their respective drug-resistant strains MRSA and MDR-*K.P.* (all isolated from patients for more vivid simulation), respectively. The mice were then treated with Omi-hyd-Dex@HA NPs at a dose of 8.0 mg/kg (including 5.0 mg/kg omiganan and 1.1 mg/kg Dex) through tail i.v. injection once every day for 4 times altogether and their survival rates were monitored. Meanwhile, vancomycin, levofloxacin, imipenem and moxifloxacin, four clinically used broad-spectrum antibiotics for treating sepsis, were respectively applied at a dose of 5 mg/kg in combination with Dex (1.1 mg/kg) to the control groups, as shown in Fig. 8A.

Among the *S. aureus*-induced sepsis models, the survival rates of the Dex + moxifloxacin group and Dex + vancomycin group were both 44.4% (4/9) in 10 d, while those of the Dex + levofloxacin group and Dex + imipenem group were lower than 22.2% (2/9), as shown in Fig. 8B. Among the MRSA-induced sepsis models, all the mice died in the Dex + levofloxacin groups, and only one (1/9) was rescued by Dex + vancomycin, Dex + imipenem or Dex + moxifloxacin (Fig. 8C). Among the *K.P.*-induced sepsis models, 33.3% (3/9) mice survived in Dex + imipenem group and not more than 11.1% (1/9) in Dex plus vancomycin, levofloxacin and moxifloxacin groups (Fig. 8D). While among the MDR-*K.P.*-induced sepsis models, none of the survival rates of the four groups was higher than 11.1% (1/9) (Fig. 8E). Therefore, the therapeutic efficacy of those four clinically used antibiotics administered in combination with Dex was limited in treating non-resistant strains and even much lower in treating drug-resistant strains. Especially, if the antibacterial spectrum of the empirical prescribed antibiotics did not cover the real pathogenic bacteria (like vancomycin on G- strains), the survival rate would be compromised. In contrast, Omi-hyd-Dex@HA NPs resulted in survival rates of not less than 66.6% (6/9) among sepsis models induced by two non-resistant strains and not less than 55.5% (5/9) among drug-resistant strain models. The contrast showed that the comprehensive protection effect of Omi-hyd-Dex@HA NPs against sepsis was much better than that of four empirically prescribed drug-combination therapy, which demonstrated the potential for clinical translation of the NPs.

Further, the main advantage of Omi-hyd-Dex@HA NPs compared with the previously reported DDSs is that they are not limited to the infection caused by certain types of pathogens, which makes them suitable for improving the current empirical treatment regimen in the clinic [56], [57], [58], [59]. Especially in the clinically isolated resistant and non-resistant bacteria-induced sepsis models, Omi-hyd-Dex@HA NPs markedly increased the survival rates of different types of infected mice, but the combinations of conventional antibiotics and Dex hardly enhanced the survival outcomes (Fig. 8), confirming the excellent therapeutic effects of Omi-hyd-Dex@HA NPs for sepsis.

## 4 Conclusion

In summary, we have fabricated Omi-hyd-Dex@HA NPs from omiganan-Dex conjugates and HA coating to overcome the limitations of empirical treatment of sepsis. The versatile nanoformulation could specifically accumulate in the infected tissues and responsively release omiganan and Dex to synergistically control the infections and inflammation response. The Omi-hyd-Dex@HA NPs possess good biocompatibility and significantly improve the survival of sepsis mice in multi-types of infected models. Thus, Omi-hyd-Dex@HA NPs might be a promising candidate for systemic sepsis management in clinical emergency treatments. Furthermore, the Omi-hyd-Dex@HA NPs display broad-spectrum therapeutic effects against diverse pathogens, including viruses, bacteria and fungi, all of which can be targeted by omiganan. This expanded scope allows for the utilization of these nanoformulations for managing other infectious diseases, such as bacterial pneumonia and tissue infections. Moreover, future optimization of the peptide component also holds the potential to further enhance their antibacterial efficacy.

## Conflicts of interest

The authors report no conflicts of interest. The authors alone are responsible for the content and writing of this article.

## Appendix Supplementary materials

Image, application 1
