# Supplementary material for: An infection-microenvironment-targeted and responsive peptide-drug nanosystem for sepsis emergency by suppressing infection and inflammation
Source: Asian J Pharm Sci. 2023 Nov 28;18(6):100869. doi: 10.1016/j.ajps.2023.100869 (PMC10755722; doi:10.1016/j.ajps.2023.100869)
Supplement: Supplementary file 1 [file mmc1.docx]

Supplementary materials

**An infection-microenvironment-targeted and responsive peptide-drug nanosystem for sepsis emergency by suppressing infection and inflammation**

**1. Supplementary materials and methods**

***1.1 Preparation and characterization of PLGA(Dex)@HA NPs, PLGA(Omi)@HA NPs and Omi-hyd-Dex@PAA NPs***

PLGA(Dex)@HA NPs and PLGA(Omi)@HA NPs were prepared using the nanoprecipitation method. Firstly, PLGA (10 mg; amino-terminal; Mw 38,000-54,000 Da) was dissolved in DMSO (4 mL). Then, 2 mg of dexamethasone or omiganan-NH-NH_2_ was added to the DMSO solution to prepare the stock solution. Under vigorous stirring, 20 mL of diH_2_O (0.4% PVA, m/V) was dropwise added. After 4 hours, another 20 mL of diH_2_O containing 5 mg of hyaluronic acid (HA) was added. The mixture was stirred for 1 hour, followed by the addition of 2 mg of EDC and 0.5 mg of Sulfo-NHS to continue the reaction for 2 hours. This allowed for the conjugation of carbonyl groups in HA with amino groups on the PLGA core, ultimately strengthening the HA coating.The nanoparticles were separated by centrifugation (15,000 rpm; 10 min) and then re-dispersed in a PBS solution containing 0.1% PVA by sonication. The particle size and zeta potential of the NPs were measured using the Brookhaven NanoBrook 90plus instrument. The drug loading content was determined by HPLC analysis after disrupting the nanoparticles with DMSO.

The fabricaiton of Omi-hyd-Dex@PAA NPs was similar to that described in section **2.4**. Omi-hyd-Dex (10 mg) and PLGA (2 mg; Mw 38,000-54,000 Da) were dissolved in 1 ml of DMSO. Under intense stirring, 15 ml of diH_2_O (0.4% PVA, m/V) was added into the DMSO solution drop by drop. After 60 minutes, another 20 ml of diH_2_O containing 5 mg of PAA was added for coating. The stable Omi-hyd-Dex@PAA NPs were produced by adding 2 mg of EDC and 0.5 mg of Sulfo-NHS for building covalent cross-linking between PAA and core NPs. After 4 hours, the resulting suspension was centrifuged at 15,000 rpm for 10 minutes and resuspended in PBS. The obtained Omi-hyd-Dex@PAA NPs were subjected to the same aforementioned characterization process.

***1.2 The analytical HPLC spectrum of Omi-hyd-Dex***

The Omi-hyd-Dex samples were subjected to purification through reverse-phase high-performance liquid chromatography (RP-HPLC) using a SHIMADZU system equipped with a C-18 column (Pntulips®; BP-C18, 5um, 4.6x250 mm; Lot: T05B18-046250). The chromatographic separation was performed at room temperature, with detection achieved at a wavelength of 225 nm using a UV detector. A gradient of 5–55% acetonitrile was utilized over a total time of 30 minutes. To introduce the peptides or derivatives onto the column, they were dissolved in dimethyl sulfoxide (DMSO) and eluted by increasing the gradient between water (with 0.1% trifluoroacetic acid, TFA) and acetonitrile (with 0.1% TFA). The eluted peptides were detected by their UV absorption, and the desired fractions were manually collected. The purity of the collected fractions, containing the desired product, was confirmed by LC-MS. The purified fractions were combined and subsequently freeze-dried.

***1.3 Serum fluorescence intensity-time profiles of FITC-labeled Omi-hyd-Dex@HA NPs***

Rats (8 weeks old) were injected with a single dose of FITC-labeled Omi-hyd-Dex@HA nanoparticles at a concentration of 10 mg/kg. Blood samples were collected at specific time intervals. After centrifugation at 800rpm for 3 minutes, the supernatant was separated. The fluorescence intensity of FITC in serum was determined using fluorescence spectrophotometry (n=3).

**2. Supplementary figures and tables:**


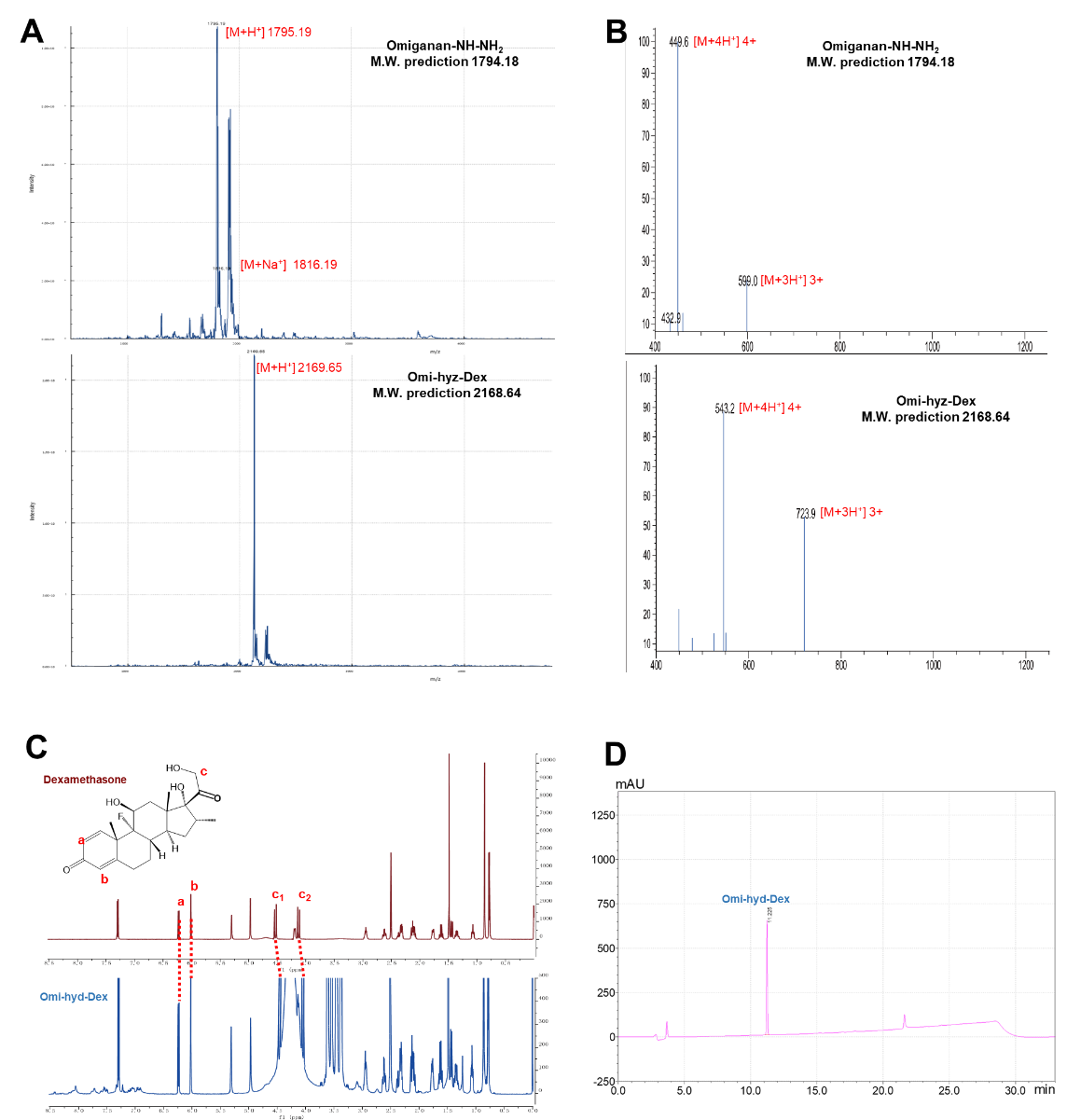


**Fig. S1. Structural and purity analysis of Omi-hyd-Dex.** A) MALDI-TOF method results of Omiganan-NH-NH_2_ and Omi-hyd-Dex; B) ESI-MS method results of Omiganan-NH-NH_2_ and Omi-hyd-Dex from GL Biochem company. C) ^1^H-NMR (600M Hz) spectra of dexamethasone and Omi-hyd-Dex in DMSO-*d*_6_. D) The analytical HPLC spectrum of Omi-hyd-Dex (product purity:94.19%).


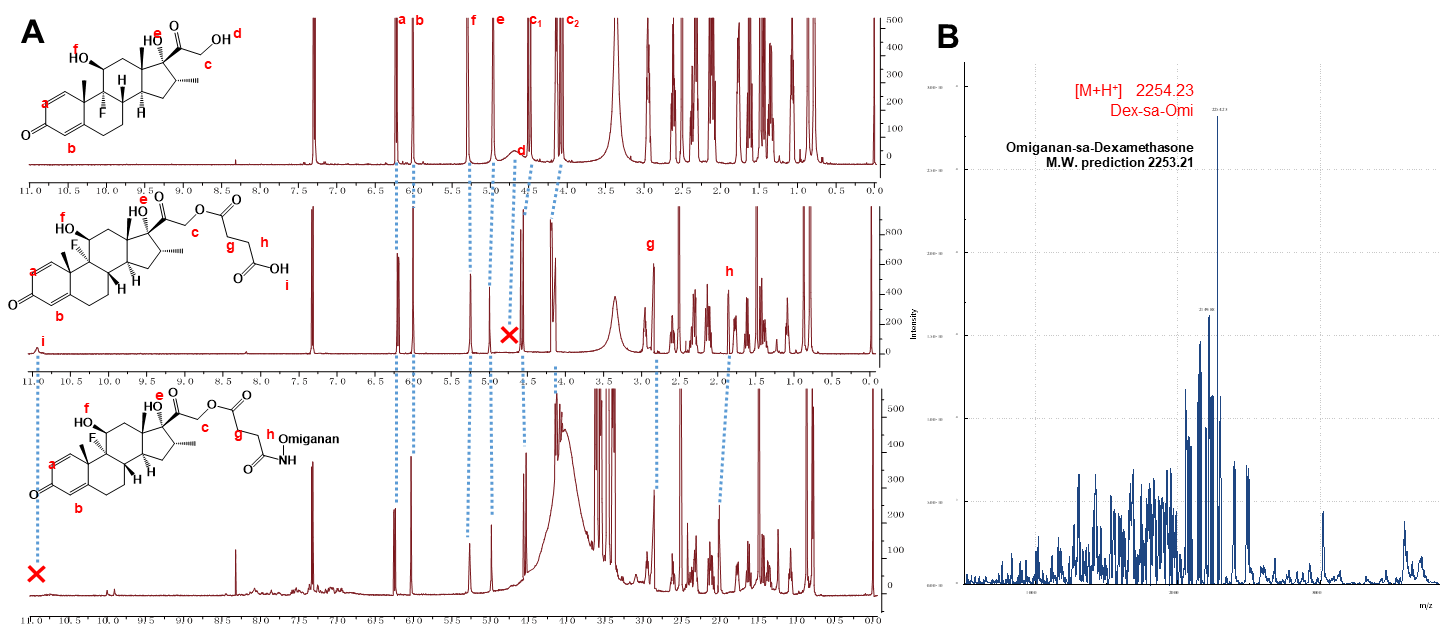


**Fig. S2. Structural analysis of Omi-sa-Dex.** A) ^1^H-NMR (600M Hz) spectra of dexamethasone, Dex-sa-COOH (dexamethasone-21-succinate) and Omi-sa-Dex in DMSO-*d*_6_. B) The mass spectrometric data of Omi-sa-Dex.


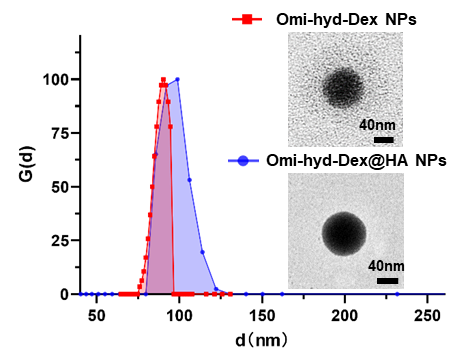


**Fig. S3. Hydrodynamic particles size (diameter) and TEM images of Omi-hyd-Dex NPs (87.3 nm) and Omi-hyd-Dex@HA NPs (96.6 nm).**


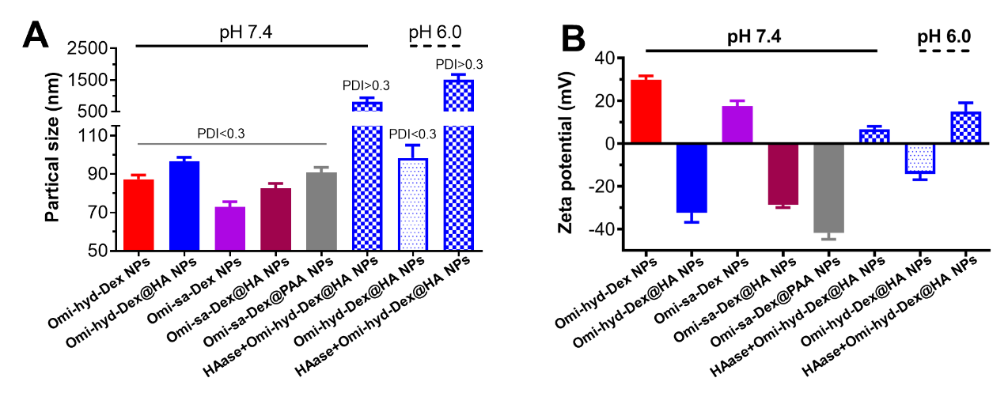


**Fig. S4. Hydrodynamic particles size (diameter; A) and surface zeta potential of NPs (0.05 mg/ml; B) under pH 7.4 and 6.0 buffer environment detected by dynamic light scattering.**

**
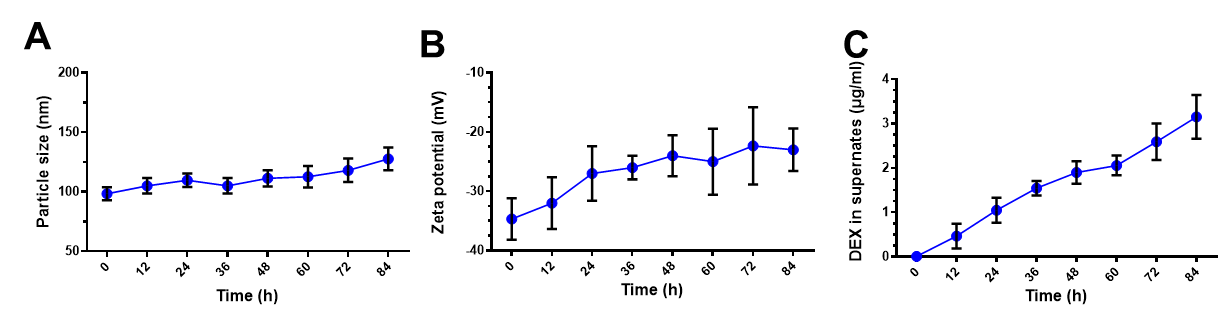
**

**Fig. S5. Physical and chemical stability of Omi-hyd-Dex@HA NPs in plasma.** Omi-hyd-Dex@HA NPs were dispersed at a concentration of 100 μg/ml in DMEM (with 40% fresh mice serum and 0.01% sodium azide). The size (A), surface potential (B), and DEX release (C) of the nanoparticles were monitored for 84 hours.

**
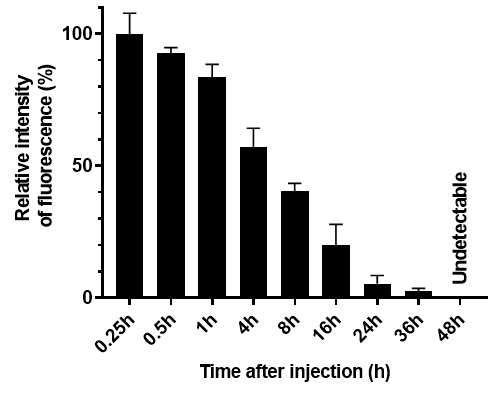
**

**Fig. S6. Serum fluorescence intensity-time profiles of FITC-labeled Omi-hyd-Dex@HA NPs *in vivo*.** One dose of FITC-labeled Omi-hyd-Dex@HA NPs (10 mg/kg) was injected into rats and blood samples were collected at specific time points. Fluorescence intensity of FITC in serum was measured by fluorescence spectrophotometers.


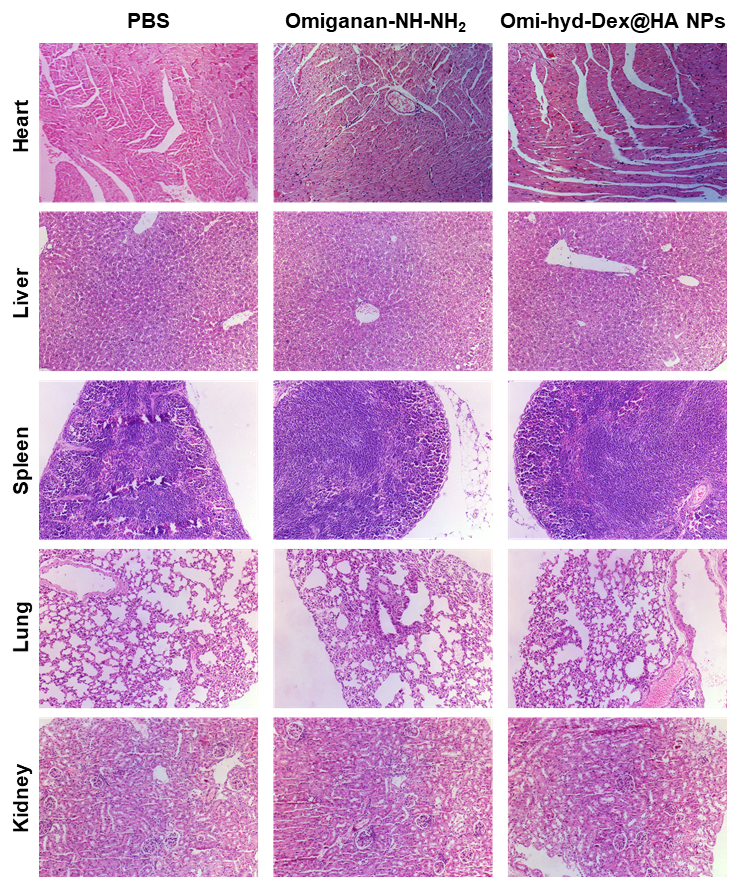


**Fig. S7. The H&E staining of major organs from mice receiving four dose of Omi-hyd-Dex@HA NPs (peptide equivalent of 10 mg/kg) did not show any obvious change compared with PBS group.**


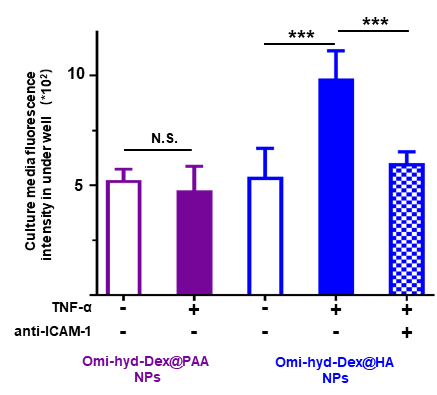


**Fig. S8. Omi-hyd-Dex@HA NPs penetrating ability assessment.** Fluorescence intensity of culture media in the lower chambers was detected after 24-h incubation. For ICAM-1 inhibition, antibody was added for 24 h before NPs incubation.


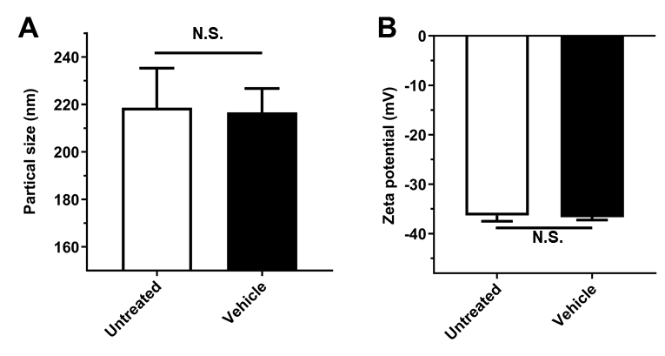


**Fig. S9.** **The vehicle (45 μL DMSO added into 2 mL LPS aggregates) did not affect the hydrodynamic size and zeta potential of LPS aggregates.** The particle size and surface zeta potential were determined by Malvern DLS device with electrode probe.


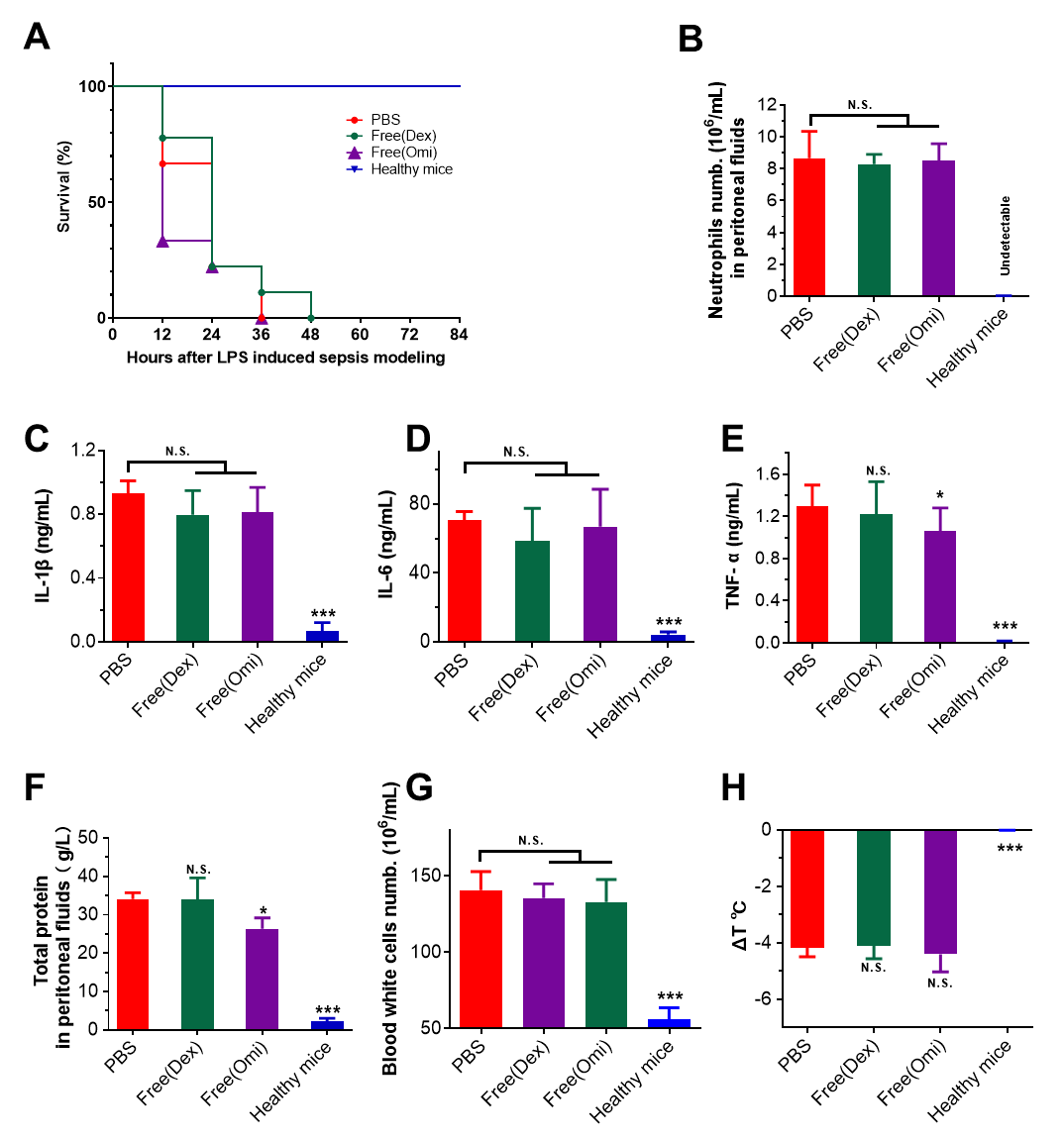


**Fig. S10. The comprehensive anti-inflammation therapeutic efficacy of free Dexamethasone and Omiganan-NH-NH_2_ *in vivo*.** (A) Experiment timeline and survival percentages (*n* = 9 per group) of lethal dose LPS-induced sepsis model mice receiving treatments. Sepsis mice treated with PBS were used as a negative control. LPS untreated mice were used as a healthy control. (B) Neutrophils number, (C-E) pro-inflammatory cytokines (TNF-α, IL-6, IL-1β) concentrations, and (F) total protein mass quantification in peritoneal fluid collected from sepsis mice receiving various treatments (*n* = 3 per group). (G-H) Blood white cells number and body temperature change one day after treatment. Compared with body temperature before modeling. Data are shown as mean ± SD, **p* < 0.05, ***p* < 0.01, ****p* < 0.001, N.S., not significant.


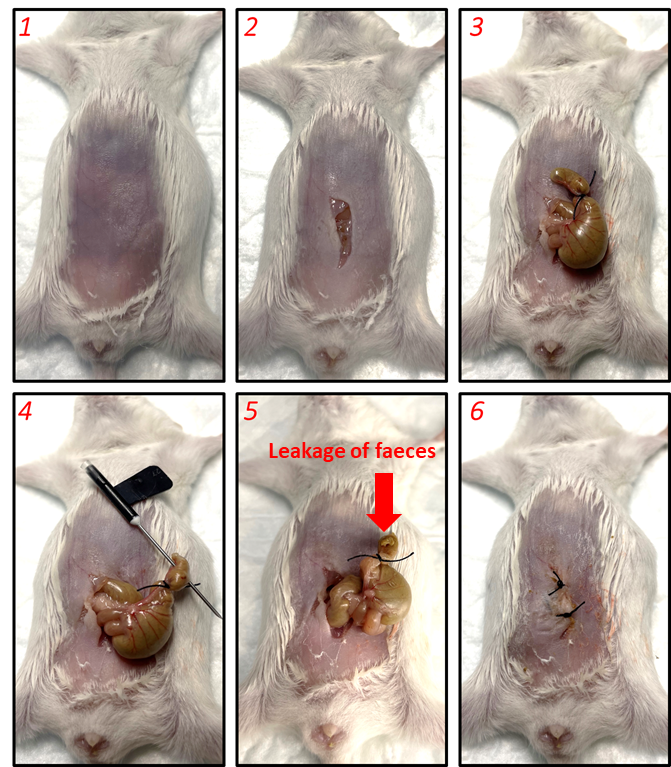


**Fig. S11.** **Images shown of cecum ligation and puncture (CLP) modeling process.** 30% of the full length of cecum was ligated and punctured with a 26 G needle for feces leakage.


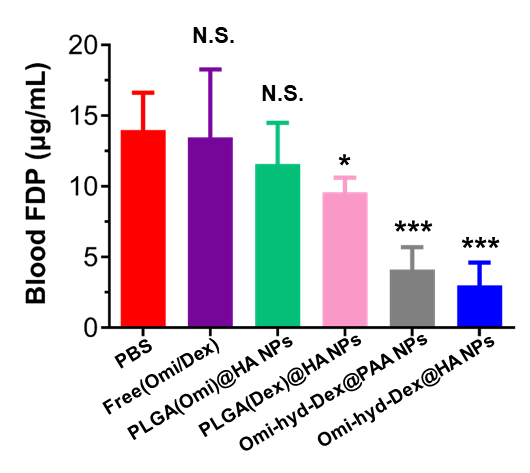


**Fig. S12.** **Blood serum fibrinogen degradation products (FDP) level detection on day 1 after treatment on CLP-induced sepsis model.**


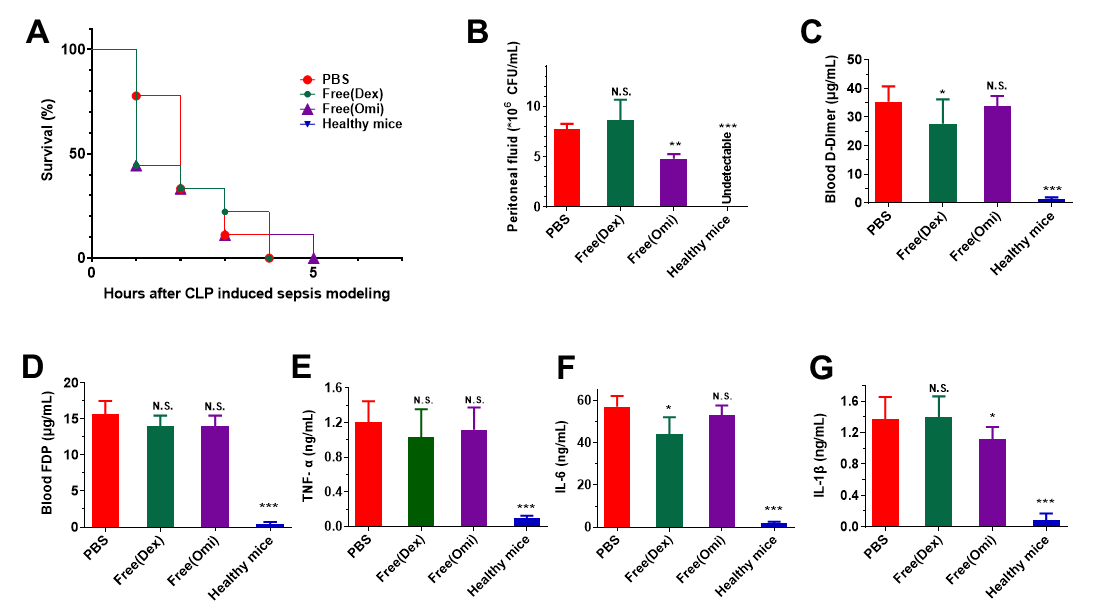


**Fig. S13. Four rounds of free Dexamethasone or Omiganan-NH-NH_2_ treatment on CLP-induced polymicrobial sepsis did not alter sepsis survival, bacterial proliferation, D-Dimer level, and pro-inflammatory cytokines production.** (A) Survival percentages (n = 9 per group) of CLP model mice receiving treatments. Sepsis mice treated with PBS were used as a negative control. Sham surgery treated mice were used as a healthy control. (B) Bacterial colonies number of peritoneal fluid inoculated on Brain Heart Infusion (BHI) agar plates in aerobic condition for 18 h. (C-D) Blood serum D-Dimer and FDP detection on day 1 after treatment. (E-G) Pro-inflammatory cytokines (TNF-α, IL-6, IL-1β) concentrations in peritoneal fluid of the mice receiving treatment. Data are shown as mean ± SD, **p* < 0.05, ***p* < 0.01, ****p* < 0.001, N.S., not significant.


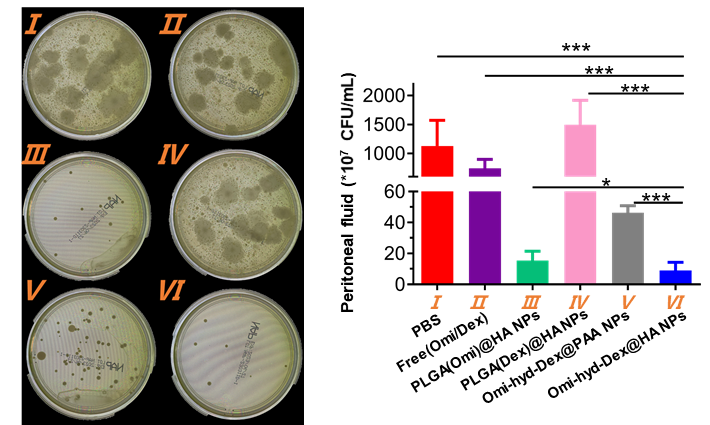


**Fig. S14. Representative images (left) and bacterial colonies number (right) of peritoneal fluid inoculated on agar plates in anaerobic condition for 36 h.** The CFU assay was applied to monitor bacterial concentration. The images present above were accomplished with diluted peritoneal fluid (1*10^-7^), while in group of PBS, free (Omi/Dex) and PLGA(Dex)@HA NPs collected peritoneal fluid needed further dilution (1*10^-10^) to obtain countable images.

**Table S1. The size and zeta potential of all used NPs (n=3) in PBS at pH 7.4.**

| **Name of NPs** | **Partical size (nm)** | **Zeta potential (mV)** |
| --- | --- | --- |
| Omi-hyd-Dex NPs | 87.3±3.85 | +29.8 |
| Omi-hyd-Dex@HA NPs | 96.6±4.33 | -32.3 |
| Omi-hyd-Dex@HA NPs (DiR-labeled) | 92.7±5.52 | -28.9 |
| Omi-hyd-Dex@HA NPs (FITC-labeled) | 98.3±3.73 | -31.4 |
| Omi-hyd-Dex@PAA NPs | 90.6±2.97 | -41.8 |
| Omi-sa-Dex NPs | 73.0±4.61 | 17.4 |
| Omi-sa-Dex@HA NPs | 82.7±3.78 | -28.7 |
| PLGA(Omi)@HA NPs | 97.4±6.94 | -27.1 |
| PLGA(Dex)@HA NPs | 90.8±5.36 | -26.1 |
